# Supplementary figures and images for: Factors influencing uptake of COVID-19 diagnostics in Sub-Saharan Africa: a rapid scoping review
Source: PLoS One. 2025 Mar 20;20(3):e0305512. doi: 10.1371/journal.pone.0305512 (PMC11925277; doi:10.1371/journal.pone.0305512)

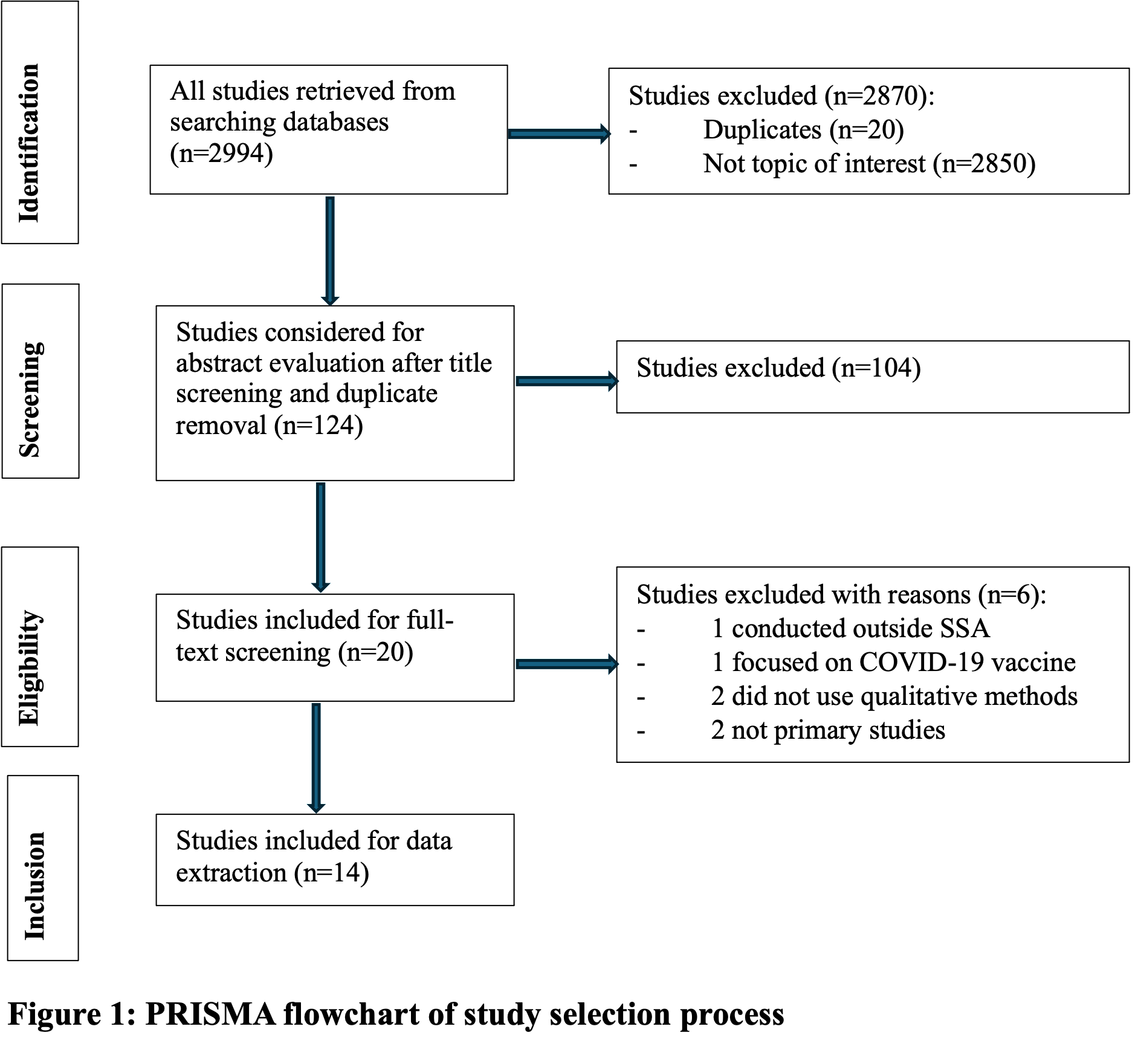

Supplement: S1_Fig — (TIF) [file pone.0305512.s001.tif]
