# Supplementary material for: Factors influencing uptake of COVID-19 diagnostics in Sub-Saharan Africa: a rapid scoping review
Source: PLoS One. 2025 Mar 20;20(3):e0305512. doi: 10.1371/journal.pone.0305512 (PMC11925277; doi:10.1371/journal.pone.0305512)
Supplement: S2_Table — (DOCX) [file pone.0305512.s003.docx]

## **S2_Table_Quality and relevance evaluation form**

| Quality and relevance questions | Study | | | | | | | | | | | | | |
| --- | --- | --- | --- | --- | --- | --- | --- | --- | --- | --- | --- | --- | --- | --- |
|  | (Amoo et al., 2020) | (Oleribe et al., 2021) | (Nxumalo et al., 2021) | (Lewis et al., 2021) | (Rispel et al., 2021) | (Schmidt et al., 2020) | (Brumwell et al., 2022) | (Asare et al., 2023) | (Ha et al., 2022) | (Asiimwe et al., 2021) | (Carlitz et al., 2021) | (Yamanis et al., 2023) | (Mohammed et al., 2021) | (Chabeda et al., 2022) |
| Abstract and title: Did they provide a clear description of the study? | 4 | 4 | 4 | 4 | 3 | 4 | 4 | 4 | 4 | 4 | 4 | 4 | 4 | 4 |
| Introduction and aims: Was there a good background and clear statement of the aims of the research? | 4 | 4 | 4 | 4 | 4 | 4 | 4 | 4 | 4 | 4 | 4 | 4 | 4 | 4 |
| Method and data: Is the method appropriate and clearly explained? | 4 | 3 | 4 | 4 | 4 | 4 | 4 | 4 | 4 | 4 | 4 | 4 | 4 | 4 |
| Sampling: Was the sampling strategy appropriate to address the aims? | 4 | 4 | 4 | 4 | 4 | 4 | 3 | 4 | 4 | 4 | 4 | 4 | 4 | 4 |
| Data analysis: Was the description of the data analysis sufficiently rigorous? | 4 | 4 | 4 | 4 | 4 | 4 | 4 | 4 | 4 | 4 | 4 | 4 | 4 | 4 |
| Ethics: Have ethical issues been addressed, and what have the necessary ethical approvals gained? | 4 | 4 | 4 | 4 | 3 | 3 | 4 | 4 | 4 | 3 | 3 | 3 | 4 | 4 |
| Results: Is there a clear statement of findings? | 4 | 4 | 4 | 4 | 4 | 4 | 4 | 4 | 4 | 4 | 4 | 4 | 4 | 4 |
| Generalizability: Are the findings of this study transferable (generalizable) to the wider population? | 3 | 3 | 4 | 4 | 4 | 4 | 4 | 4 | 4 | 4 | 4 | 4 | 4 | 4 |
| Implications and usefulness: How important are these findings to policy and practice? | 4 | 4 | 4 | 4 | 4 | 4 | 4 | 4 | 4 | 4 | 4 | 4 | 4 | 4 |
