# Supplementary material for: Factors influencing uptake of COVID-19 diagnostics in Sub-Saharan Africa: a rapid scoping review
Source: PLoS One. 2025 Mar 20;20(3):e0305512. doi: 10.1371/journal.pone.0305512 (PMC11925277; doi:10.1371/journal.pone.0305512)
Supplement: S3_Table — (DOCX) [file pone.0305512.s004.docx]

**S3_Table.docx_Study selection and data extraction**

| Author/publication year | Country | Study design | Population and sample size | Conclusions |  |
| --- | --- | --- | --- | --- | --- |
| Amoo et al., 2020 | Nigeria | Mixed-methods using in-depth interviews and quantitative survey | 27 interview participants (healthcare workers) and 1030 survey participants (members of the public) | The test strategy although largely successful, is largely dependent on Internet penetrability. In low- and medium-income countries where internet penetration is not yet optimal, establishing this test strategy as the sole modality of testing may disenfranchise a large proportion of the populace from participating in the test. Other limitations include the possibility of inadvertently promoting inequity in sample collection in the community and, the fact that only those who have cars are likely to present at the drive-through platform despite the introduction of a walk-in component. Only 22% of participants were in the walk-in group. These limitations, therefore, mean that this testing modality will be best utilized as an adjunct to the conventional mode of testing and not as a substitute. |  |
| Oleribe et al., 2021 | Nigeria | Online qualitative survey using semi-structured interviews | 495 adult (18 years and above) members of the public | COVID-19 has resulted in a global great paradigm shift. Three important priorities set the pace for a post-COVID-19 reality check and these are: planning for future pandemics through the procurement and supply of essential healthcare products, including PPE and specific training of frontline medical personnel; strengthening crisis management and response through harmonisation of regional pandemic management committees to be coordinated at a national level and greater provision of healthcare funding for public health awareness campaigns. As economic restrictions are gradually eased to reopen personal and business activities, the possibility of facing public disquiet over persistent policies, such as social distancing in the “The New Normal” are a real possibility unless mass education is undertaken at all levels from radio, television and importantly in 2021, social media platforms. Although difficult to extrapolate findings from other countries, as commonly held beliefs vary from country to country [9], a recent Italian questionnaire held that young people were more likely to comply with social control measures if they had incorporated physical activity of some form into their daily lives at home during the lockdown period. Future Nigerian questionnaires should look at this issue acting as a mitigating factor in the general public dissatisfaction that we have found in our survey, given that exercise alleviates boredom and provides a sense of well-being, even during lockdown [10]. Steps to return to a robust economy in the peri COVID era would include maintained handwashing, social distancing and limited group contact until COVID-19 vaccines are widely available in Nigeria. We advocate coordinated forward planning for public safety until the concept of vaccination is widely accepted by the Nigerian general public, rather than being viewed with suspicion. Policymakers need to be adaptable to rapidly changing conditions, given fluctuating case numbers and mounting fatality rates. The question of international travel is also an issue for policymakers, given that people these days have been used to freedom to travel wherever they choose. However, it is known from other viral diseases that travel serves to spread cases around the world, and COVID-19 had been no different in this respect [1]. Public opinions on limiting international travel also need to be assessed in the Nigerian context in future studies. |  |
|  |  |  |  |  |  |
| Nxumalo et al., 2021 | South Africa | Qualitative using semi-structured interviews | 15 primary healthcare practitioners | The findings of this study reveal that perceptions and understanding regarding COVID-19 amongst primary healthcare practitioners were mainly centred on a perception of fear, which was related to both known and unknown factors regarding the outcomes of illness associated with the pandemic. The findings also reveal that participants’ perceptions of the pandemic were mainly associated with misinformation received through social media. Overall, the findings of this study reveal that primary healthcare practitioners’ perceptions and understanding of COVID-19 are generally negative. This is cause for concern because it implies that these healthcare workers may be unable to provide the optimum quality of healthcare services as required at a primary care level. Furthermore, these healthcare workers are most at risk for developing mental health issues because of the lack of accurate information regarding the outbreak. This could potentially compromise health practitioners’ levels of job satisfaction potentially disrupting service delivery. |  |
| Lewis et al., 2021 | South Africa | Qualitative using online open-ended questionnaire | 60 diagnostic radiographers | Radiographers in Gauteng, SA are exposed to the highest number of COVID-19 infections in SA. They have experienced changes to staff allocations and work-hours as well as the implementation of stringent infection control and social distancing measures. Moreover, they face mental, physical, emotional and financial challenges. However, honing the positives, they have initiated strategies towards resilience. |  |
| Rispel et al., 2021 | South Africa | Qualitative using key informant interviews, and document analysis | 36 key informants (incl. policy makers, healthcare workers, advocacy groups) | [Our case study found that there were missed or wasted opportunities to invest in primary health care as the foundation of the health system, partner with communities and civil society, and to explore relationships or collaboration with the private health sector. These missed opportunities reflect longstanding and unresolved areas of contention. For example, the National Disaster Management Act makes provision for the state to make regulations or issue directions for the purpose of assisting and protecting the public, providing relief to the public and/or dealing with the effects of a disaster [34]. However, government did not consider any regulations to ensure greater equity between the public and private health sectors or one national response.](applewebdata://A2FCB1B6-153A-448A-9B45-509C1F9E6EFA/../../../Zotero/storage/8JCG3H2U/article.html#pone.0261339.ref034) |  |
| Schmidt et al., 2020 | South Africa | Qualitative using interviews | 60 participants (community members, civil society group members, private sector representatives) | Stigmatizing responses to Covid-19 revealed that specific groups (i.e. ‘black’ South Africans) perceive themselves to be immune to catching the virus, whilst blaming other groups for being responsible (and more vulnerable) for the transmission of the disease (i.e. ‘white’, wealthy South Africans and people of Asian descent). Developing an online collaborative platform between scientists and the South African public, to debunk myths and misconceptions about Covid-19 isintegral to maintaining preventive measures and correct knowledge about who is most vulnerable to Covid-19. Such an online collaborative platform between scientists and the South African public, should be expanded across Africa for engagement that makes use of language that isinclusive, emphasizing that viruses do not target specific groups of people or ethnicities. Terminology guidelines that makes considerate use of language should be developed with scientists, civil society representatives and representatives of specific groups of people or ethnicities in order to decrease the possibility of stigmatizing specific groups of people. Finally, communication content should be developed to further inform the public health response to Covid-19 using HSRC data to ensure itisaccessible to policy makers and wider communities of national and local policy actors, in government and civil society. |  |
| Brumwell et al., 2022 | South Africa | Qualitative using semi-structured interviews | 52 COVID-19 self-testing decision makers (health workers, civil society representatives, self-testing implementers) | South Africans are generally positive about the concept of self-testing for SARS-CoV-2 infection. For any roll-out of self-testing to be successful, trusted stakeholders, institutions, and community members, including lay people, will be required. These include government institutions, local pharmacies, private healthcare providers, and public health NGOs. In planning this roll-out, the scepticism that urban South Africans have toward government systems and institutions, particularly regarding the COVID-19 response, must be addressed. Focus should be placed on young people and vulnerable population groupings, as they have expressed concern about the various information platforms they are exposed to and their confusion about what information they should be paying attention to. Taking guidance from previous public health campaigns, civic groupings need to partner with the government in the implementation of these campaigns. The possibility of self-testing offers new opportunities for South Africans; a prospective way of increasing faith in the government and the reach of public health initiatives. In a country plagued by extremely high unemployment rates, low levels of trust in government, and insufficient healthcare-seeking behaviours, SARS-CoV-2 self-testing offers real transformative potential for a range of public goods. |  |
| Asare et al., 2023 | Ghana | Qualitative using focus group discussions | 39 COVID-19 contact tracers | We identified challenges associated with COVID-19-related contact tracing in the Bono region of Ghana while unearthing opportunities that exist for improved future contact tracing from the perspective of contact tracers. Hence, health authorities addressing these challenges and harnessing these identified opportunities could help improve contact tracing in the region in the future. |  |
| Ha et al., 2022 | Ghana | Qualitative using semi-structured interviews | 20 COVID-19 testing key informants (policymakers, implementers, frontline health workers) and community members | As vaccines are rolled out, testing will continue to play a vital role in controlling COVID-19. The main reason is that testing, followed by contact tracing and isolation of those with positive test results, will promptly allow health professionals to monitor the dynamics of the pandemic. Moreover, according to our findings, COVID-19 testing is still of particular importance to effectively controlling the transmission of the virus in Ghana. Most of the participants confirmed that testing, as an important prevention measure, should be secured with adequate resources and stable health systems. Also, good health governance and leadership, effective resource management, and digitalized information system are successful factors influencing extensive COVID-19 testing. However, upscaling testing capabilities and facilities is faced with several bottlenecks, such as uneven resource distribution, COVID-19 infodemic, and constraints to service delivery. From the analysis, multilateral cooperation and joint partnerships with diverse stakeholders will play a critical role in facilitating active community participation, investment in GMP, and multilateral political commitment in taking bold actions to build strategies to respond to emerging pandemics. Also, a new research area, HPSR, will be a stimulus for many countries to restructure and develop stronger health systems for future pandemics. |  |
| Asiimwe et al., 2021 | Ghana | Qualitative using semi-structured interviews | 27 COVID-19 response team members (contact tracers, supervisors) and case contacts | The study concludes that the contact tracing was generally perceived to be helpful in COVID19 containment in Ghana. However, adhering to self-quarantine protocol had many challenges for both contact tracers and the contacts. Improving coordination and quick release of test results to contacts isnecessary for COVID-19 containment. Lastly, the provision and supply of Personal Protection Equipments and motivation needs to be addressed to help position the country well for effective contact tracing. Measures should be taken to enforce quarantine among COVID-19 contacts to minimize spread to family, work colleagues and community members. |  |
| Carlitz et al., 2021 | Tanzania | Qualitative using in-depth interviews | 40 participants (public healthcare workers, social welfare organisations, village leaders) | To date, the literature on COVID-19 containment and mitigation (and on government responses to disease outbreaks more generally) has focused primarily on explaining variation across rather than within countries. However, given decentralization reforms worldwide, much of the work of disease management is carried out at the local government level. As such, focusing on the central government level may limit our understanding of whether and how policies are implemented. Whereas prior studies have blamed policy-implementation gaps on street-level discretion, we show how local officials “make policy” that can help fill a void of central government leadership. In highlighting the experiences of local bureaucrats, we provide an important perspective that is often missing from the literature on health politics and policy, particularly studies of low-income settings. Documenting the challenges whether and how policies are implemented. Whereas prior studies have blamed policy-implementation gaps on street-level discretion, we show how local officials “make policy” that can help fill a void of central government leadership. In highlighting the experiences of local bureaucrats, we provide an important perspective that is often missing from the literature on health politics and policy, particularly studies of low-income settings. Documenting the particular challenges local officials face, and adaptations they make in light of those challenges, contributes to a small but growing literature on the role of local bureaucrats in management and policy implementation in the global South. Our study suggests that scaling up the capacity of and resources for street-level bureaucrats to manage a public health emergency, especially in countries with limited central government response, may be highly strategic. Local officials face, and adaptations they make in light of those challenges, contributes to a small but growing literature on the role of local bureaucrats in management and policy implementation in the global South. Our study suggests that scaling up the capacity of and resources for street-level bureaucrats to manage a public health emergency, especially in countries with limited central government response, may be highly strategic. |  |
| Yamanis et al., 2023 | Tanzania | Qualitative using in-depth interviews | 56 participnts (healthcare workers, social welfare organisations, village leaders) | Our study is the first to reveal public health SLBs’ perceptions of the factors that challenged and facilitated mass COVID-19 vaccination in Tanzania. Similar to other studies based on surveys of African citizens, 23–26 our findings revealed that community perceptions, particularly misinformation/disinformation, and inadequate health system investments were significant challenges to COVID-19 vaccine uptake. Adding to this, we found that the persistence of the denialist views of Tanzania’s deceased president was a significant obstacle. On the other hand, facilitating factors included executive branch endorsement of vaccination, greater transparency regarding COVID- 19 information, global and regional integration, the availability of mobile clinics and community-based campaigns to disseminate health information. In light of the need to scale up COVID-19 vaccination in Africa and our findings, below we present recommendations for improving vaccine uptake. |  |
| Mohammed et al., 2021 | Ethiopia | Qualitative using semi-structured interviews | xx participants (COVID-19 prevention task force members, healthcare workers, community members) | Before and after the first Corona case was confirmed on June 16,2020, certain preparatory and response measures were taken in Woreta. These include, the establishment of a task force and other supportive technical and enforcement agents. The established bodies have taken measures in early March to enforce stay at home and physical distance by restricting movement, closing various institutions and markets. But the measures failed and reversed since they were not well planned and coordinated. Other preparatory and response measures including the existence of a rapid response team, the safety practice of health workers, the conduct of sample collection activity as well as surveillance and social assistance campaigns were relatively good beginnings in Woreta. But weak and declining enforcement, loose coordination, discontinuous risk communication, politicization, unsupportive logistic and supply chain were weaknesses.The low preventive practice of residents manifested by non-compliance to public health and social measures was another weakness. In addition, underequipped health system identified by absence of COVID-19 diagnostic laboratory, functional quarantine and isolation centers, shortage of nurses, basic supplies, and PPEs was a gap that marked low preparedness in the health setting. So, there should be a political commitment and coordinated engagement of all stakeholders grass roots level**.** Although this study was conducted prior to the development of anti-Covid-19 vaccines, the pandemic's infliction upon residents is not culminated with the development such treatment. So, more task needs to be done for an effective response. In this regard, the study is expected to have a twofold importance: first, it will assist the local government to evaluate its covid-19 readiness and fill the operational and resources gaps. Second, it will help the health workers to design a local strategy for community engagement and bringing behavioral change. |  |
| Chabeda et al., 2022 | Kenya | Qualitative using semi-structured interviews and focus group discussions | 50 COVID-19 self-testing stakeholders (providers, implementers, and advocacy groups) | In conclusion, key decision-takers believe that the implementation of SARS-CoV-2 self-testing in Kenya would be acceptable by the general population, who would welcome community-grounded, time- and cost-saving patient empowerment initiatives. However, decision-takers shared a number of key critical factors that need to be in place for feasible and valuable COVID-19 self-testing in Kenya. These included support from regulatory authorities, post- and pre-test counselling interventions, and mass awareness campaigns for the public and health practitioners to combat stigma around COVID-19 and financial burden of having to self-isolate following a reactive result. Ensuring these measures are in place will enable members of the Kenyan public to safely and effectively use SARSCoV-2 self-tests once they are approved by Kenyan authorities. |  |
