# Supplementary material for: Factors influencing uptake of COVID-19 diagnostics in Sub-Saharan Africa: a rapid scoping review
Source: PLoS One. 2025 Mar 20;20(3):e0305512. doi: 10.1371/journal.pone.0305512 (PMC11925277; doi:10.1371/journal.pone.0305512)
Supplement: S1_Text — (DOCX) [file pone.0305512.s005.docx]

**S1_Text.docx_**Study ethics approvals

Ethical approval numbers for in country 3ACP studies, informed through this scoping review.

Nigerian ethic approvals: “3ACP-Nigeria: Enhancing access to COVID-19 rapid tests and self-testing":

1. Federal Capital Territory Health Research Ethics Committee (FHREC), approval number FHREC/2022/01/29/09-03-22

- WHO ERC (CERC.0165)
- London School of Hygiene and Tropical Medicine (26886).

1. Zimbabwean ethic approvals: An evaluation of different use cases for COVID-19 antigen self-testing in Zimbabwe-a mixed methods study”

- Medical Research Council of Zimbabwe (MRCZ) Ref A2872
- London School of Hygiene and Tropical Medicine Ref 26931
- WHO ERC CERC.0160

1. Malawian ethic approvals: “
