# Supplementary material for: Factors influencing uptake of COVID-19 diagnostics in Sub-Saharan Africa: a rapid scoping review
Source: PLoS One. 2025 Mar 20;20(3):e0305512. doi: 10.1371/journal.pone.0305512 (PMC11925277; doi:10.1371/journal.pone.0305512)
Supplement: S2_Text — (DOCX) [file pone.0305512.s006.docx]

**S2_Text.docx_**Nvivo coded data

[Files\\Included articles\\A qualitative study to explore primary health care practitioners' perceptions and understanding regarding the COVID-19 pandemic in KwaZulu-Nata South Africa](159cbec6-a3f7-40b1-8588-60b5c87fd03e)

9 references coded, 2.26% coverage

References 1-3: 0.15% coverage

The findings of this study suggest that primary health care practitioners generally have negative perceptions and understanding regarding the pandemic because of misinformation obtained from social media.

Reference 4: 0.27% coverage

information and was inherent in the fact that they were unaware of how the disease would eventually manifest if and when it arrived in South Africa. The following statements were made by participants in this regard: ‘It was terrifying in that it included sudden death, and the statistics from other countries as well – so it was frightening then.’ (P01, male, Clinical nurse practitioner)

References 5-6: 0.36% coverage

Pandemic perceptions Participants described their perceptions of COVID-19 when the pandemic occurred in South Africa which appeared to be shaped by an array of factors related to the information they received from various sources, such as the general media, social media, health circulars and peer-to-peer word-ofmouth. These perceptions were also shaped by primary and secondary experiences related to the occurrence of the pandemic in South Africa together with the health systems’ response to the pandemic.

Reference 7: 1.09% coverage

Perceptions regarding the nature and outcomes of the disease Participants described their view of the pandemic in terms of the clinical manifestation and ultimate complication of the disease, which they perceived to be a severe disease and that would ultimately lead to death. Certain participants also compared the COVID-19 outbreak with the other outbreaks such as the Ebola virus and H1N1 (commonly known as swine flu). The perception regarding COVID-19 as being deadly was a predominant perception stemming from information that had been circulating on social media before the outbreak occurred in South Africa. This was supported by the following statements: ‘When they talked about it, they talked about it as if it was similar to H1N1 [swine flu], which is the swine flu, right? it was the swine flu, mode of spread being, the mode of spread being cough droplets, yea, something of that nature. But, it was more defined as if it was a certain type of COPD [chronic obstructive pulmonary disease] or severe, or presented as some kind pneumonia and it was very deadly.’ (P05, male, Nutritionist) ‘I knew it was a viral infection that was caused by the COVID-19 virus [coronavirus disease 2019]. It was first identified in China. Yeah and that it spreads through respiratory droplets so like easily transmitted.’ (P08, male, Clinical associate) ‘I understood it to be a contagious viral flu-like illness that appeared to be strangely deadly because of the number of people that were sick and dying in countries like China and Italy as a result.’ (P12, female, Professional nurse)

References 8-9: 0.39% coverage

Misperceptions regarding coronavirus disease 2019 Healthcare workers in this study revealed that they had a distorted view of the disease. They reported that their views were related to myths about the disease that had been circulating on social media, which created and perpetuated the notion that the COVID-19 disease was not an outbreak but rather it was manufactured in a laboratory for killing people in order to control population density. Other misperceptions were related to beliefs about power relations between the governments of different countries.

[Files\\Included articles\\Challenges and opportunities for improved contact tracing in Ghana- experiences from Coronavirus disease-2019-related contact tracing in the Bono region](ba548f54-6213-4aef-bb9c-9acbb476c240)

2 references coded, 0.62% coverage

Reference 1: 0.59% coverage

Poor public education on COVID-19 Nearly half (18) of the participants highlighted that inadequate information about the disease made contact tracing difficult. It was revealed that most contacts lacked proper health education at the initial stage of the disease outbreak and this influenced their reception to contact tracing. One discussant narrated: During the first wave of COVID-19, contact tracing was difficult because most people did not get enough information with regard to the disease. People who were contacts with a positive case did not understand the reason why they had to be quarantined for you to come and pick samples. You get to a contact’s home and the person is already gone instead of staying in the house. He/she is already out into the public. Either he is gone to work, a funeral or any occasion. So, the first phase of COVID-19 contact tracing

Reference 2: 0.03% coverage

was very difficult. (GC, female 39 years old)

[Files\\Included articles\\Coping_with_Denialism_How_Street_Level_B](26f35c7b-11ff-47f2-8cd6-03ed4e1113b5)

1 reference coded, 2.15% coverage

Reference 1: 2.15% coverage

Communication Respondents unanimously agreed that their public education activities were their most effective adaptations. Although the MoHCDGEC-issued SOP mentionedcommunity education as important for epidemic response, it did not detail speciﬁc strategies(MoHCDGEC2020). Our respondents felt that collaboration with community leaders was critical for delivering effective public health education. Respondents described transmitting COVID-19 Tanzania COVID-19 Lessons 1005 Downloaded from http://read.dukeupress.edu/jhppl/article-pdf/46/6/989/1382580/989carlitz.pdf by guest on 17 November 2021 1006 Journal of Health Politics, Policy and Law prevention information via leaﬂets and posters, mobile phones, local radio, public announcements (loudspeakers), and songs. Interviewees also stressed the importance of reaching places such as motorcycle depots, markets, and places of worship. Interviewees felt that these efforts engendered a positive response from citizens, changed community attitudes about risk, enhanced adherence to mask wearing and social distancing, and lowered their fear for seeking COVID-19 treatment. They reported that the use ofloudspeakers was very effective, as citizens socializing in the streets were easily reached. Respondents also felt that collaboration with volunteers and organizations, such as international NGOs and Tanzanian businesses, was key to the success of their public education efforts. Moreover, the use of ﬁnes, where applied, enhanced citizen cooperation. Respondents described promoting public awareness of COVID-19, drawing on their experience managing other diseases. As a village ofﬁcer stated: “We have sufﬁcient experience in managing big diseases like HIV, cholera . . . so why should we be afraid of corona? This is a normal disease just like fever that we live with every day.” Many health ofﬁcials also saw their role as ﬁrst and foremost to lower psychological distress. Lowering psychological distress was mentioned in the MoHCDGEC-issued SOP (MoHCDGEC 2020). The SOP mentions that the psychosocial epidemic response teamshould reduce stigma and discrimination toward individuals withCOVID-19 and should provide psychosocial support to those affected. As such, one hospital response ofﬁcer saw their role as “reminding ...the general public that corona is not like Ebola and can be managed with minimum wear of protections by health workers.” Ward ofﬁcers engaged district ofﬁcials to educate the public and lower stigma of individuals and COVID-19 isolation and treatment units. Moreover, regional ofﬁcers used mass media and local political/reli- gious leaders to educate the public. Ward ofﬁcers reported collaborating with health ofﬁcials to provide public health education on the risks of the novel coronavirus and how citizens could protect themselves. One ward ofﬁcer said they worked together with religious leaders so that the public would understand the dangers of large groups attending burials, a traditional practice. Several ward ofﬁcers used donated PA systems with microphones to educate the public, and some used the radio. Public educationwas often done in collaboration with district ofﬁcials who provided expert-written health communication leaﬂets on coronavirus prevention

[Files\\Included articles\\COVID-19 Testing- A Qualitative Study Exploring Enablers and Barriers in the Greater Accra Region, Ghana](c3ce4fa7-5196-43e0-939f-6d855a03ba02)

4 references coded, 3.50% coverage

Reference 1: 0.80% coverage

Health Communications Public health communication plays an important role in protecting public health during pandemics. According to the interviews, the government of Ghana eﬀectively provided information through various public media for the people, particularly by educating them on measures that need to be undertaken to contain the spread of the virus. The participants asserted that mass media campaigns on adherence to health protocols and regular updates on COVID-19 by the president and health authorities positively inﬂuenced community members to adhere to health directives. A researcher from the University pointed out, “There are several TV and radio advertisements, and they are compelled to educate people. The advertisements ensured that concepts and materials on COVID19 testing are seen as culturally relevant and well understood by people.”

Reference 2: 0.86% coverage

Misconceptions About COVID-19 and Testing Almost all the participants pointed out misconceptions about COVID-19. They emphasized that some people hesitate to get tested because they consider COVID-19 as just common ﬂu. Furthermore, some people believe in rumors that coronavirus does not exist. According to our ﬁndings, these factors inﬂuenced the decision of community members to be tested for COVID-19. “Some people believe in fake news and misinformation that the virus is not real, so vaccination and testing are unnecessary. Also, July 2022 | Volume 10 | Article 908410 Ha et al. Qualitative Study: Factors Inﬂuencing COVID-19 Testing in Ghana they think COVID-19 is just like ﬂu, and big countries are using it for political purposes”—A Community Leader 1. “I have a patient who strongly believes that the virus does not exist. So, I think educating them to adhere to health protocols using TV and radio is essential.”—A Nurse 1

Reference 3: 0.53% coverage

Furthermore, the participants narrated that most women in the community do not have the same level of knowledge or information about the virus as men and how to adhere to health protocols. A senior oﬃcer at MoH cited, for example, that the level of educated men is higher compared to women, “Education will make a lot ofdiﬀerence in the heart ofpeople concerning the COVID-19 and its right information. However, at the same time, I feel that illiteracy among women can be a barrier, especially in rural areas. Unfortunately, they (women) have fewer opportunities to be educated than men.”

Reference 4: 1.31% coverage

As vaccines are rolled out, testing will continue to play a vital role in controlling COVID-19. The main reason is that testing, followed by contact tracing and isolation of those with positive test results, will promptly allow health professionals to monitor the dynamics of the pandemic. Moreover, according to our ﬁndings, COVID-19 testing is still of particular importance to eﬀectively controlling the transmission of the virus in Ghana. Most of the participants conﬁrmed that testing, as an important prevention measure, should be secured with adequate resources and stable health systems. Also, good health governance and leadership, eﬀective resource management, and digitalized information system are successful factors inﬂuencing extensive COVID-19 testing. However, upscaling testing capabilities and facilities is faced with several bottlenecks, such as uneven resource distribution, COVID-19 infodemic, and constraints to service delivery. From the analysis, multilateral cooperation and joint partnerships with diverse stakeholders will play a critical role in facilitating active community participation, investment in GMP, and multilateral political commitment in taking bold actions to build strategies to respond to emerging pandemics. Also, a new research area, HPSR, will be a stimulus for many countries to restructure and develop stronger health systems for future pandemics.

[Files\\Included articles\\Implementation of a modified drive-through sampling strategy for SARS-CoV-2-the Nigerian experience](5a4904fa-bf54-46ed-8954-5f5dfa243c8b)

1 reference coded, 1.28% coverage

Reference 1: 1.28% coverage

Suggestions and Recommendations: overall, knowledge and awareness of COVID-19 are still low and there is a need to educate people, provide counsel, and increase the uptake of the testing. The majority of the clients stated that the test was scary and invasive however, the process and turnaround time was effective and prompt. They suggested that the following strategies should be explored: 1) The test is invasive and painful and other alternatives of testing should be explored. 2) Knowledge and awareness of COVID-19 is low. NIMR should The Pan African Medical Journal. 2020;35 (Supp 2):107 | Olufemi Samuel Amoo et al. 3 educate people and create awareness at different levels. 3) Counselling should be done to reduce anxiety and distress. 4) Few people know about the centre in NIMR, awareness could increase the number of persons tested.

[Files\\Included articles\\Misconceptions, south africa](d853fdac-8274-483c-8330-22c1886f0950)

23 references coded, 8.81% coverage

Reference 1: 0.15% coverage

We found that false information circulated on social media not only instigated confusion, fear and panic, but also contributed to the construction of misconceptions, othering and stigmatizing responses to Covid-19.

Reference 2: 0.14% coverage

The study findings bring attention to the importance of developing communication materials adapted to specific communities to help reduce misconceptions, othering and stigmatization around Covid-19.

Reference 3: 0.55% coverage

Knowledge of Covid-19 and awareness ofwho is most vulnerable Study participants generally understood that Covid-19 is a respiratory disease, as is clear in the excerpt below from an interview conducted with a traditional leader. “I know that Corona is a flu-like virus. It is kind ofa fever that attacks your lungs. Ifyour immune system is compromised, you are at risk ofcontracting it. It is transferred through droplets and ifmaybe you touch a place, where somebody that is infected by it may have coughed and there were droplets that were there. Ifyou touched there and then touch your face, any opening on your face, there is a risk ofcontracting it. I think for you to be safe you [must] wash your hands, cover your nose and keep a distance from other people. I think in a nutshell that is what it is” (Male, traditional leader: Gauteng)

Reference 4: 0.37% coverage

Similarly, study participants’ awareness ofrisks was also highlighted in terms ofthe follow- ing: Physical contact with those who are infected, be it outside their homes, in public transport, or by touching contaminated surfaces like the money that is passed around by passengers in a minibus-taxi. Awareness ofpreventive measures are demonstrated in the following quotations: “I’ve heard about it yes, that it’s contagious, you have to cover your mouth, there’s no handshake involved. Ifyou cough, you use your elbow and you must always use sanitizer after

References 5-6: 0.57% coverage

everything that you do. Keep your surfaces clean like the door handle, [keep] everything clean, keep on bleaching or [using] Jik [i.e. bleach] or anything else. The lockdown you [must] stay indoors. I think it’s for 21 days in order to avoid getting in touch with people, in case somebody has it on the outside so if you’re indoors you will be safe from getting the virus” (Female, sex worker: Gauteng) “Yes I use public transport. . .with public transport you have no choice, I mean you are in one [minibus] taxi obviously you have to pass someone the money or someone has to pass the money to you. I just try not to touch my face and nose, as soon as I get where I am going, I make sure I wash my hands, because I do not have gloves. I use a mask because we got masks at work. In a [minibus] taxi; I also have the sanitizer for my hands as well” (Female, person with disabilities: Western Cape)

References 7-8: 0.57% coverage

tudy participants were aware that older people and those with immunocompromised conditions are more vulnerable to catching the virus (see Table 2): “It would be my mother-in-law because she is in her 80s. She is in an old-age unit, they are on lockdown. They can’t even come up and have their breakfast and lunch. So she’s the one who I am mostly concerned about” (Female, person with disabilities: Gauteng) “I am taking it seriously because I live with my gran and she’s a 67-year-old lady, so they are more vulnerable to this disease” (Female, out ofschool youth: KwaZulu-Natal) “The way that I understand it [for] people who are living with HIV and TB, that it’s very dangerous for them, so people [who] live with these illnesses that it can also cause your death and so [I am concerned about] myselfwho is HIV-positive” (Female, person living with HIV: Western Cape)

Reference 9: 0.53% coverage

Covid-19 false information, myths and misconceptions In the weeks following the announcement ofthe lockdown, the South African government announced the implementation ofa mass door-to-door screening and testing campaign. At the same time, false information was also distributed on social media warning South African communities to refrain from testing (see Table 3). “Now, they have people around, testing. The internet says; don’t allow the testing and then the other people said they should allow the testing. So that for me is [the] very confusing part. So, I don’t know what to do if people are doing door-to-door testing. You do not know whether to welcome them or to check them or chase them away. Then some people say don’t open the door [for] them, they will give you the Coronavirus and so that’s where

Reference 10: 0.16% coverage

Yes, I think you saw the guy arrested yesterday, a video circulating on Whatsapp a man saying they test with contaminated testing kits. People must not test, now we don’t know what is the truth” (Male, person with disabilities, Western Cape)

Reference 11: 0.14% coverage

“. . .The other [thing that they are] say[ing] [is], don’t accept any test for Corona because it’s already infected” (Female, relief aid worker providing services to migrant communities: KwaZulu-Natal)

Reference 12: 0.44% coverage

False information distributed on social media also caused confusion, fear and panic amongst South African communities. In some instances, healthcare workers reported that the distribution of false information influenced public responses to the screening and testing campaign: “Like as clinic staffwe go in door- to-door, there are incidences where a house owner would refuse for us to go in, saying we don’t want your vaccines because they have Corona. Then we had to explain that we are not injecting people, we are just screening and asking questions. People are really scared, because ofwhat they heard. . .” (Female, community health worker: Western Cape)

Reference 13: 0.29% coverage

Moreover, false information about the availability ofa Covid-19 vaccine also circulated on social media. Study participants mentioned that in the communities in which they live, rumors spread ofa vaccine that was released for Covid-19: “Another myth is that there is a vaccine. I do not know whether that is true, but apparently there is a vaccine and people need to be vaccinated for Coronavirus” (Female, old age home carer: Gauteng)

Reference 14: 0.30% coverage

In addition to false information about the availablity ofa Covid-19 vaccine, home remedies, such as drinking hot water, was seen as a cure for the disease: “We drink hot water. Some videos said that we must try and drink hot things. Ifyou come into contact with the virus and [it’s] still in your throat you can drink water. It is possible that it can be washed into the stomach and the stomach will kill the virus” (Male, community health worker: KwaZulu-Natal)

Reference 15: 0.68% coverage

Parallel to false information ofthe availablity ofa Covid-19 vaccine, were findings suggest- ing that eating the right things, in this instance maize (corn) meal porridge might serve as a protective factor to Covid-19: “I also believe that people in our country, or in Africa doesn’t see the severity of it, they believe that they are immune from it. Just as an example, I was in a Uber, before the lockdown started and I made a conversation about [Covid-19] and I asked the driver is he worried about getting infected. And so his response to me was Oh no I won’t get the virus because I eat pap [translated from Afrikaans, (i.e. local language) to English, means porridge, referring to maize meal traditionally made]. And I ask him what do you mean and he says, because my system is strong it will only get people who don’t eat pap, who are weak.So eating pap, the whole perception ofbeing healthy is misconstrued there in terms ofpap for some people pap is a healthy lifestyle” (Transgender woman: Sexual and gender minority group member, Gauteng)

References 16-17: 0.67% coverage

Inasmuch as qualitative findings point to study participants having correct knowledge and awareness regarding the transmission and prevention ofCovid-19, myths and misconceptions about the disease was also evident in the data (see Table 4). In the first instance, study participants revealed that young people do not see themselves at risk ofbecoming infected with the virus that causes Covid-19. Hence, they raised concerns that the youth were not taking the Covid-19 pandemic seriously: “My issue is that the younger ones are not taking it seriously. Even though it will mostly kill the elderly since their immune system is compromised, the young and middle aged are not taking it seriously. They think it is one ofthose things that will just pass but the most vulnerable and most at risk are the elderly and the young ones because their immune system is still weak, it is compromised. Those are the people who are often sick. But it is mostly going to affect and kill elderly people” (Male, faith based leader: Gauteng)

Reference 18: 0.48% coverage

Myths that the virus is man-made, originating from “eating the wrong things” and transmitted via 5G technology were also observed in the data: “I’ve heard quite a lot, very confusing, some legit, some not legit. Basically it’s a virus that attacks your lungs and flu-like symptoms. Some people think that its man-made, some people think that it’s due to people eating the wrong things!” (Female, out ofschool youth: KwaZulu-Natal) “Ok let me start about the 5G. . .I keep asking myself, how can I get infected with 5G. . .how do I get infected by it. Because I believe I only get it by touching someone who has the virus and then maybe I will touch my face you see. So this thing of5G. . .” (Female, person living with disability: Western Cape)

Reference 19: 0.06% coverage

Myths and misconceptions regarding the zoonotic origin ofCovid-19 also emerged from the data:

Reference 20: 0.89% coverage

Myths Misconceptions about who is most vulnerable to Covid-19 “I know that it cannot survive in [a] hot environment it requires a cold environment and it also spread[s] through saliva” (Male, taxi driver: Gauteng) “People first thought that it was the virus of the sick. Especially those people who are living in the rural areas and the notion that the virus does not necessarily attack people that are ‘black’ in the name, so that was the attitude ofmany people, but also lack ofinformation and arrogance of our people” (Male, traditional leader: Gauteng) “Just thought that if I’m not an old person if I haven’t travelled overseas that means that I wouldn’t get it that was the case” (Pregnant Woman: Gauteng) “So, for me that’s fake but it’s been going around in videos and voice notes. There was also something that I heard [that] it’s not attacking, ‘black’ people, ‘black’ raced people and stuff like that, that’s what I’ve been hearing” (Female, out ofschool youth: KwaZulu-Natal) “There [are] people saying oh no, this sickness don’t affect ‘black’ people!” (Female, relief aid worker for migrant communties, KwaZuluNatal) “I’ve got some friends in Eastern Cape, who don’t [abide] by the rules and they always making fun about we won’t get coronavirus it is for ‘whites’ only so they think it is a joke” (Male, person with disabilities: Western Cape)

Reference 21: 0.29% coverage

“I even heard that the virus is associated with bats and snakes” (Male, community health worker: KwaZulu-Natal) “Ay, ja I saw in the media, social media, it was yesterday I think [it] was a ‘white’ man, I think it was in the hospital, there was something like a worm from [his] lip it was a doctor taking out the worm from the lip they called it, it’s a Coronavirus, I will forward it to you. . .”” (Female, person with a disability: KwaZulu-Natal)

Reference 22: 0.84% coverage

Misconceptions that people ofa higher socio-economic status were perceived to be more vulnerable to SARS-CoV-2 than others appeared to be common across key informant interviews. Having travelled overseas, race and class were constructed as protective factors against Covid-19. Claims that the disease is a “white-man’s disease”, were influenced by assertions that those assigned ‘black’ in South Africa are naturally immune to the disease. “There is a perception that again it is a ‘white’ person disease because ‘white’ people, and I say that in inverted commas are, the ones who travel overseas and they brought it to Africa. Again my perception is there is a lot ofknowledge and understanding that it’s not about ‘white’ people who also travel ‘black’ people also travel, when we first found out about the first lot ofpatients in KwaZulu-Natal. I know that one ofthe people who has gone overseas was an African woman in my mind are these people not educating themselves enough or do they just block out oftheir mind that this was an African woman who went there. There perhaps it becomes a class thing, lower class people don’t look at it as if they would [because it is] upper class people who travel a lot” (Transgender woman, sexual and gender minority group member: Gauteng)

Reference 23: 0.70% coverage

“At first, I’m [going to] sound racist but they claim that only people who are ‘white’ will be infected by people who are mostly interacting with [‘white’ people]. Plus, that only ‘white’ people can get infected” (Pregnant woman: Gauteng) “Some people used to say that it can’t affect ‘black’ people. They thought it was for ‘white’ people. Ja that was mostly [what was said] out here that it doesn’t affect ‘black’ people, we have strong genes and so on” (Male, community health worker: KwaZulu-Natal) “People are taking it as a joke, it’s lockdown but people are still all over. They only think it will affect the old people with chronic diseases alone, not the young, youth. They don’t want to wash [their] hands, [they] don’t want to cover when one is coughing or sneezing, it’s supposed to be a lifestyle thing not to do it because there is Corona. They also believe it was not meant for Africans, it was for China and Italy and all those stories” (Female, community health worker: Western Cape) Such misconceptions informed othering and stigmatizing responses to Covid-19.

[Files\\Included articles\\Preparedness and response to covid-19 in Woreta Town, North West Ethiopia](bf44178e-777e-489a-ba3d-c59766ae3cd6)

7 references coded, 1.79% coverage

References 1-3: 0.79% coverage

Risk communication and community engagement Awareness creation and communication activities were done inside and outside the health center using different mech- anisms such as; home visits, leaﬂets, loud speaker-loaded cars, inﬂuential persons (religious leaders and political leaders), scheduled teachings arranged for patients and their care givers. In addition, public shows were made in which health workers have travelled in procession along the main street to transmit COVID-19 prevention message. Similarly, a mask-wearing show was held by governmental workers including the police, the health professionals and other civil servants. Apart from the above, people were getting information by their own effort from national TV, radio, telecom voices, or from social media accounts. However, the communication tasks were less fruitful since misunderstandings, groundless rumors, suspicions and denials are still prevalent. According to kebele 01 and 03 administrators, the main challenge for prevention was the politicized outlook and negligence against the government’s Covid-19 response activities.

Reference 4: 0.16% coverage

The way the positive tested person was hold was not persuasive. The person was confused to have the virus without any symptom and failed to accept the result. It was the ﬁrst conﬁrmed case in the town detected in bus station on 16 June 2020

Reference 5: 0.22% coverage

“Everyone knows about Covid-19, being told always. These days, there is no talk other than Covid-19 in every house and in the media. Those who distribute false rumors are just deliberately politicizing. My reservation lies only in the continuity of awareness creation activities here”, a high school teacher

Reference 6: 0.35% coverage

“Our health teams perform a show, we taught six times by microphone, I personally participated in public announcement, 3000 leaﬂets are distributed holding health messages, the mayor “There are continuous campaigns for the last ﬁve months, measles campaign, family health campaign and covid-19 campaign. so, we have no time for sitting in oﬃce. Moving door to door and informing to wash hands, to use mask, and to report suspected cases for the kebele administrator.” A health extension worker in kebele 01

Reference 7: 0.27% coverage

and residents were seriously following this case. Parallel to this development, there was a rumor that laboratory results of Covid-19 cases were consciously fabricated to turn the public attention away from the hot political issues. Since the rumor was quickly disseminated, some people dared to deny its existence while others concluded that Covid-19 was not a serious health threat.

[Files\\Included articles\\Self testing for COVID 19 in Durban and Eastern Cape South Africa a qualitative inquiry targeting decision takers](6ca4769f-dd80-4c55-b8ad-484b514bf85b)

2 references coded, 0.74% coverage

Reference 1: 0.34% coverage

Also described as ‘apathy’ among poorer groups toward the disease, this is more likely a manifestation of general lack of knowledge about COVID-19, the test, and appropriate actions to be undertaken if an individual believes they are sick. To many, all SARS-CoV-2 prevention and testing protocols and regulations felt very diﬃcult to comprehend. As a result, the public’s ability to adhere to them was described as uneven.

Reference 2: 0.40% coverage

Information campaigns were thought necessary to increase public awareness of self- testing availability, to build knowledge of how self-testing should be used, and to instruct the public on what actions should be performed following a reactive result. Radio, television, social messaging, and social media were commonly recommended means for spreading this information. However, some HCWs and RCSs were adamant that selftesting be delivered alongside information campaigns.

[Files\\'Better to die of disease than die of hunger'- the experience of Igwes (traditional rulers) in the fight against the COVID-19 pandemic in rural South-east Nigeria](4b886fd6-2d7d-4db8-a3f7-e828c98da09c)

2 references coded, 0.92% coverage

Reference 1: 0.37% coverage

Many of the traditional rulers who acknowledged that their rural communities are at risk of an outbreak nevertheless believed that God would protect them. As stated earlier, Nigerians are religious and dependent on God, especially when they feel they are powerless in situations. The traditional rulers cannot force their subjects to adhere to set measures; therefore, they turn to God for protection

Reference 2: 0.55% coverage

Disbelief about the existence of COVID-19: Analysis of the data showed that many rural people do not believe in the existence of the disease and this makes it difficult for the traditional rulers to ensure their safety. The traditional rulers argued that the Igbo ethnic group are very stubborn and would only believe in the existence of the disease when they witness increased mortality as a result of infections: As we say, Igbo fụ na-anya fa ekwe na ụka [Igbos don’t believe until they see], anyi anaghi agba ọsọ na abughi mgbe ọmazuru anyi arụ [we don’t run from the rain until it drenches us]. (P2

[Files\\Included articles\\Challenges and opportunities for improved contact tracing in Ghana- experiences from Coronavirus disease-2019-related contact tracing in the Bono region](ba548f54-6213-4aef-bb9c-9acbb476c240)

2 references coded, 0.96% coverage

Reference 1: 0.41% coverage

These include inadequate personal protective equipment, harassment by contacts, politicisation of the discourse around the disease, stigmatization, delays in processing test results, poor remuneration and lack of insurance package, inadequate staffing, difficulty in locating contacts, poor quarantine practices, poor education on COVID-19, language barrier and transportation challenges. Opportunities for improving contact tracing include cooperation, awareness creation, leveraging on knowledge gained in contact tracing, and effective emergency plans for future pandemics.

Reference 2: 0.55% coverage

Politicisation of the discourse around the COVID-19 outbreak The politicisation of the discourse around the disease was another challenge encountered by almost all contact tracers, as it was passionately reported by each group. Some Asare et al. BMC Infectious Diseases (2023) 23:335 Page 5 of 14 contacts requested the political affiliations of contact tracers before accepting to cooperate. This was because they believed that the government had an ulterior motive for how the disease was managed. A discussant in District E remarked that: Politicising the disease is a challenge to us [contact tracers]. This is because you will get to a contact’s home and they start to politicise the entire process [of contact tracing] and they begin to ask you which party you belong to. (GE, male, 43 years old)

[Files\\Included articles\\Coping_with_Denialism_How_Street_Level_B](26f35c7b-11ff-47f2-8cd6-03ed4e1113b5)

3 references coded, 5.06% coverage

Reference 1: 0.56% coverage

Surveillance In March 2020 the MoHCDGEC issued a series of standard operating procedures (SOPs) that included surveillance for COVID-19. The guidelines stated that all patients with acute respiratory illness should be screened for COVID-19 and that health care facilities, especially emergency centers and outpatient clinics, should identify an appropriate area and person to do the screening (MoHCDGEC 2020). Health facilities were advised to use a national screening checklist, which included symptoms, travel history, and contact with probable or conﬁrmed cases. Travelers, especially those from countries with COVID-19 outbreaks, were to be quarantined for 14 days and tested. However, it is important to note that on May 3, 2020, President Magufuli suspended the head of the national laboratory, just before stopping all COVID-19 surveillance

Reference 2: 1.95% coverage

Challenges and Adaptations in the Context of Hegemonic Party Rule As noted above, while the ruling CCM party has dominated national politics since independence, opposition parties have begun to make inroads at the local level. We therefore investigated whether and how the challenges faced by local health ofﬁcials and corresponding adaptations varied between opposition- and CCM-led councils, and more generally how local ofﬁcials responded to high-level denialism. Overall, we do not ﬁnd that political party control at the local level affected the nature of challenges reported. Furthermore, praise for President Magufuli among our interviewees was widespread and did not seem to vary as a function of local political leadership. Limited Impact of Local Political Control While ofﬁcials in some opposition districts expressed criticism of the government’s limited response to COVID-19, we did not ﬁnd much evidence to suggest that local political control played a signiﬁcant role in determining local responses. For instance, theDMO in one opposition-led district felt that the government needed to do a better job ofcommunicating the severity of the disease, so as to make their job easier: “[I would like to] request the government to continue insisting members of general public to take precautionary measures to manage corona, despite reopening of schools and borders. That will help health workers who are giving education about how to manage corona.” Downloaded from http://read.dukeupress.edu/jhppl/article-pdf/46/6/989/1382580/989carlitz.pdf by guest on 17 November 2021 1008 Journal of Health Politics, Policy and Law The lack ofautonomy in procurement was noted by the DMO in another opposition-led district. This ofﬁcial went on to express some frustration with the central government’s downplaying of the seriousness of COVID19, citing “a need of avoiding political interference on health issues.” The ofﬁcial went on to say, “The government move to reassure its people that the disease does not exist in Tanzania . . . has made people to live carelessly without taking any precautions and this could lead to new infections and serious impacts.” However, such frustration was registered in some CCM-led districts as well, with one district health ofﬁcer lamenting “high ofﬁcial leaders’ statements about reported positive cases of animals and fruits and discouraging the use ofmasks.” This statement likely refers in part to a speechMagufuli made in early May, in which he suggested the country’s caseload was overstated as a result of “compromised” test kits resulting in false positives. The president described samples that were taken from farm animals and a papaya and labeled with human names. He claimed the nonhuman samples tested positive, presenting this as evidence that labs were falsifying positive test results. Opposition politicians have suggested this statement was part of a broader campaign to minimize the scope of the pandemic in advance of the election (Peralta 2020).

Reference 3: 2.55% coverage

Praise and Acquiescence: Toeing the Party Line On the whole, criticism of the ofﬁcial response was limited among our respondents. Rather, our interviewees lauded the president’s role in reducing fear and stigma. For instance, the health secretary in one CCM-led district noted, “we learned from our top leaders like President John Pombe Magufuli that fear can bring greater impacts than the disease itself.” A street-level ofﬁcer in another CCMdistrict echoed this and went on to say that treating COVID-19 as a “normal” disease meant “we avoided economic threats like hunger and stagnation ofother developmental activities.” Such statements were not limited to ruling-party strongholds, how- ever. As a street-level ofﬁcial in an opposition-held district noted, “Top government ofﬁcial leaders’ statements like the president help to lower fears and challenges in managing corona.” Award-level ofﬁcial in another opposition district stated, “Our president decided not to close borders with other countries, allowing all economic activities to take place and reopening of universities and schools. This helped citizens to see corona disease as normal as other diseases.” Ofﬁcials at the regional level also lauded Magufuli’s role in lowering fear among the general public. Suchwidespread praise Downloaded from http://read.dukeupress.edu/jhppl/article-pdf/46/6/989/1382580/989carlitz.pdf by guest on 17 November 2021 Carlitz, Yamanis, and Mollel - Tanzania COVID-19 Lessons 1009 for Magufuli may reﬂect the fact that the majority of our interviewees were appointed rather than elected ofﬁcials and rely on the central government for their salaries. Beyond praising the president, local ofﬁcials in both ruling-party strongholds and opposition-led districts expressed support for traditional remedies—in line with Magufuli’s advice on disease management. As oneward ofﬁcer in Morogoro explained: “We can be independent when dealing with pandemics like corona by using traditional ways, prayers and alternative treatments such as kujifukiza (steam inhalation), the use of lemons, and drinking tangawizi (ginger).” In Arusha, the other more rural region, one ward ofﬁcer articulated support as follows: “We should also strive to have innovative solutions for treatment and vaccines for COVID-19 using our local remedies instead of depending on everything from other countries.” This echoed Magufuli’s characterization ofWestern public ofﬁcials and donors as “imperialist” in calling for Tanzania to follow the WHO guidelines and international best practices (Patterson and Balogun 2021). Nevertheless, one district medical ofﬁcer in Arusha disagreed with the use of natural remedies, stating, “The use oflocal remedies like kujifukiza should be an alternative, not a priority, treatment in response to corona disease. [We should] only use medical treatments from health institutions.” In the more urban regions of Tanzania, there was less uniform support for traditional remedies as treatment for COVID-19. In Dodoma, one ward ofﬁcer who was against their use stated, “Traditional remedies without proper guidelines might threaten human life because in our ward there was one death caused by traditional remedies where the person’s skin and internal organs burnt.” In Dar es Salaam, one ward ofﬁcer and two district ofﬁcers commented that traditional remedies should be included as part of the package of tools to “help manage” the novel coronavirus, but they were less emphatic about their use as a sole treatment for COVID-19. More generally, these responses illustrate how local ofﬁcials adapt pragmatically in the context of authoritarian governance—and, particularly, a context of high-level denialism. Our interviewees did not appear to see their actions as going against the president. Rather, they tended to interpret his statements as helping reduce fear and allowing life to go back to normal

[Files\\Included articles\\COVID-19 Testing- A Qualitative Study Exploring Enablers and Barriers in the Greater Accra Region, Ghana](c3ce4fa7-5196-43e0-939f-6d855a03ba02)

2 references coded, 1.50% coverage

Reference 1: 0.21% coverage

Better health governance through political leadership, community participation, multisectoral collaboration, effective resource management, and information systems played a crucial role in catalyzing COVID-19 testing.

Reference 2: 1.29% coverage

Political Leadership The study revealed how eﬀective leadership from the Government of Ghana positively inﬂuenced eﬀorts to foster COVID-19 response strategy. Many participants conﬁrmed the important role of political leadership in enhancing the general understanding of the Ghanaian population about the COVID-19 pandemic and response mechanisms, such as adhering to health regulations and getting tested. They reported that the president’s guidance strongly inﬂuenced a sense of collective responsibility and encouraged people to adhere to response Frontiers in Public Health | www.frontiersin.org 4 strategies, particularly on being tested against the virus. More importantly, the participants emphasized that leadership and coordination between the government and the local authorities fostered a sense of collective responsibility and timely responses to the threats posed by the pandemic. “We received lots ofhope from the government and the president. Our president was really keen on tackling the pandemic, and, with a lot ofsupport from other governmental entities, such as the Ghana Health Service and the National Commission for Civic Education (NCCE), we could respond to the COVID-19 pandemic timely. We were highly encouraged to get tested by the president, and his leadership uplifted the motivation ofgetting tested.”- A researcher frommedical research institute 1.

[Files\\Included articles\\Decision-takers_Attitudes_Towards_SARS-CoV-2_Self](bfbb18b7-0c58-4768-9f45-b90bd8eb782a)

1 reference coded, 0.33% coverage

Reference 1: 0.33% coverage

ome rural RCSs cited the “Bill Gates conspiracy” and “other conspiracy and religious theories” as reasons why healthcare professionals could not reach all people who should be tested. ”The myths, the myths and misconceptions that this coronavirus is, is associated with like the 666 that is the anti-Christ.“ (Female RCS, 53 years old, Taita Taveta)

[Files\\Included articles\\Diagnostic radiographers’ experience of COVID-19, Gauteng South Africa](3ee8a49c-e68a-4298-a8e2-435fee6c4e53)

2 references coded, 2.76% coverage

Reference 1: 2.16% coverage

Radiographers describe being sad, terriﬁed, confused, stressed, scared, exhausted, anxious, overwhelmed, frustrated, uncertain and panicked. Their “roller coaster” ofmixed emotions has stirred up an “emotional war” draining them emotionally, physically, ﬁnancially and mentally.33e35 Seeing patients out of breath, deteriorate, being ventilated and dying; in the same way, seeing colleagues falling ill and some dying, take a toll on radiographer's mental health. Radiographer's mental health is marauded even further by not being able to see family and friends coupled with the anxiety of contracting and transmitting the disease.33e35 There is also the notion that healthcare workers are not human and therefore should cope.28 These descriptions are reﬂected in the following quotes: “Emotionally, physically and mentally exhausting …. I'm tired of seeing patient's condition deteriorate because there is no cure so their symptoms are only being treated. I'm tired of seeing patients lose their lives. It's also heart breaking to see patients so S. Lewis and F. Mulla Radiography 27 (2021) 346e351 short ofbreath and a lot ofthe patients are elderly and end up on ventilators …” R10 “We are so proud of you!! Great Job!! That's what the frontliners hear. But have you ever looked beyond that … to the homes and extended family of those that are on the frontline?… nobody on the outside considers the emotional impact of this on the immediate and extended family … entire family dynamics and values have been uprooted……Are we not human as well? Do we not have feelings and fears? Do you assume that when you qualify with a health degree our hearts automatically turn to steel …. WE ARE HUMAN just like you” R12

Reference 2: 0.59% coverage

“No longer can I just reach out and give you a hug or comforting shoulder to cry on … your safety and mine … distance … invisible barriers we need to create” R12 Yet some radiographers report being told that “staff” are over- reacting. During the SARS epidemic radiographers were motivated through the crisis by reassurance.57 “I, for one know that at work, we were often told “staff is overreacting” so I fought this and I felt a lot of emotional and mental strain on myself” R51

[Files\\Included articles\\Innovations, contestations and fragilities of the health system response to COVID-19 in the Gauteng Province of South Africa](a637b471-34ef-4fe8-899e-0f210e67bcc1)

2 references coded, 0.72% coverage

Reference 1: 0.25% coverage

Several key informants commented positively on the visible and strong political leadership by the Gauteng Premier and the former Member ofthe Executive Council (MEC) for Health. We were led properly in our response. The Provincial Command Centre had given us clear, accurate instructions on what needs to be done e.g. the setting up ofquarantine centres. It showed bold leadership (KI 8, Central Hospital Manager). Two key informants from civil society organisations also echoed the strong, visible political leadership. One said:

Reference 2: 0.47% coverage

The Premier has given the impression ofefficiency and transparency, and that has inspired confidence, and the beliefthat everything was under control. I was impressed with the slides presented at the media briefings, and the attempt at showing hard data (KI 23, Civil Society). Several key informants reported that the pandemic provided an opportunity for innova- tions at various levels ofthe health system. In one health district, one key informant reported that contact tracing started early March 2020 when the country was in hard lockdown, with very few reported COVID-19 cases. The district health team developed written standard operating procedures. They established two types oftracer teams: physical tracers (primarily nurses) whose responsibility was to visit the homes ofCOVID-19 cases to test and educate other members ofthe households, and telephonic tracers (lay counsellors or other tracers), who were responsible for monitoring the cases and contacts every day for the 14-day isolation period.

[Files\\Included articles\\Misconceptions, south africa](d853fdac-8274-483c-8330-22c1886f0950)

3 references coded, 1.53% coverage

Reference 1: 0.30% coverage

In addition to false information about the availablity ofa Covid-19 vaccine, home remedies, such as drinking hot water, was seen as a cure for the disease: “We drink hot water. Some videos said that we must try and drink hot things. Ifyou come into contact with the virus and [it’s] still in your throat you can drink water. It is possible that it can be washed into the stomach and the stomach will kill the virus” (Male, community health worker: KwaZulu-Natal)

Reference 2: 0.68% coverage

Parallel to false information ofthe availablity ofa Covid-19 vaccine, were findings suggest- ing that eating the right things, in this instance maize (corn) meal porridge might serve as a protective factor to Covid-19: “I also believe that people in our country, or in Africa doesn’t see the severity of it, they believe that they are immune from it. Just as an example, I was in a Uber, before the lockdown started and I made a conversation about [Covid-19] and I asked the driver is he worried about getting infected. And so his response to me was Oh no I won’t get the virus because I eat pap [translated from Afrikaans, (i.e. local language) to English, means porridge, referring to maize meal traditionally made]. And I ask him what do you mean and he says, because my system is strong it will only get people who don’t eat pap, who are weak.So eating pap, the whole perception ofbeing healthy is misconstrued there in terms ofpap for some people pap is a healthy lifestyle” (Transgender woman: Sexual and gender minority group member, Gauteng)

Reference 3: 0.54% coverage

Othering and stigmatizing responses to Covid-19 Misconceptions about natural immunity to the virus further informs a discourse ofothering and stigmatization. Qualitative data revealed that people ofAsian descent were blamed for the transmission ofthe disease: “Also a lot ofpeople said it was from the Chinese people, the virus comes from them, [be] cause ofwhat they eat and all ofthat” (Transgender woman, sexual and gender minority group member: Gauteng) “It’s going to be stigma towards them. Like the time ofHIV. People who are [living with] HIV are still stigmatized in our society. So this is the same thing with the Coronavirus. In Gugulethu (name ofa township in Cape Town) mall, after people found out that the virus was from China [they stopped] going to [the] China store” (Female, shebeen patron: Western Cape)

[Files\\Included articles\\Perceptions and opinions of Nigerians to the management and response to COVID-19 in Nigeria](05196e70-6ed3-4244-806c-4e0bd5d8c317)

2 references coded, 1.59% coverage

Reference 1: 0.93% coverage

“Government should rise up to their responsibilities”: several respondents believed that the government had failed the Nigerian people, did not provide good leadership in the fight against COVID-19, delayed closure of the borders, did not properly enforce social distancing and “stay at home” measures, did not educate the general public appropriately to know more about the epidemic and the preventive steps to take, and did not properly equip healthcare facilities, or train healthcare workers to control the outbreak. Respondents therefore called for responsible leadership, proactive and deliberate steps by Government and its agencies towards COVID-19 control and prevention in Nigeria.

Reference 2: 0.65% coverage

“Depoliticising the epidemic”: some respondents believed that the epidemic was politicized. To this end, they asked for depoliticization of the COVID19 control process. One said, “We may not have the luxury of large isolation centres to cater for all the people who will test positive, but we can make the treatment protocols available in every locality, so that the majority of people can be treated in their homes. This should be possible if testing kits are available for early detection.”

[Files\\Included articles\\Preparedness and response to covid-19 in Woreta Town, North West Ethiopia](bf44178e-777e-489a-ba3d-c59766ae3cd6)

4 references coded, 1.53% coverage

Reference 1: 0.73% coverage

Coordination In Woreta, three major units of actors were organized for Covid-19 prevention and control which include a Task Force, a Technical Committee and a Rapid Response Team (RRT). The task force was a multi-sectoral force drawn from heads of various oﬃces. It was led by the Mayor and has lower branches. But there were variations in the makeup of task forces in the lower branches. The technical committee was composed of seven professionals working in Woreta Health Center, a governmental facil- ity. The committee was led by the center’s head and undertaken technical tasks jointly with the RRT. Both of them were supportive structures for the task force. The taskforce had a political leverage that it could use in the town to mobilize the community in to pandemic prevention and mitigation. But its effort was restricted in this regard. The following complain was forwarded by a health extension worker “The authorities and the police used to support our COVID-19 campaigns at the initial period of outbreak but now the support is interrupted.”

Reference 2: 0.31% coverage

According to kebele 01 and 03 administrators, the main challenge for prevention was the politicized outlook and negligence against the government’s Covid-19 response activities. “When you tell them the right thing (to do or not do something) about Covid-19, they try to associate it with politics or certain selﬁsh beneﬁts of leaders and falsify your advice. Or else they accept the advice without implementing it”, acting 01 kebele administrator

Reference 3: 0.21% coverage

Politicization and intentional negligence “When you tell them the right thing (to do or not do something) about Covid-19, they try to associate it with politics or certain selﬁsh beneﬁts of leaders and falsify your advice. Or else they accept the advice without implementing it” 01 kebele administrator

Reference 4: 0.27% coverage

and residents were seriously following this case. Parallel to this development, there was a rumor that laboratory results of Covid-19 cases were consciously fabricated to turn the public attention away from the hot political issues. Since the rumor was quickly disseminated, some people dared to deny its existence while others concluded that Covid-19 was not a serious health threat.

[Files\\Included articles\\Self testing for COVID 19 in Durban and Eastern Cape South Africa a qualitative inquiry targeting decision takers](6ca4769f-dd80-4c55-b8ad-484b514bf85b)

3 references coded, 0.94% coverage

Reference 1: 0.41% coverage

The possible harms caused by a positive self-test were considered to mirror the harms that could follow a positive provider-initiated test. Potential negative consequences of a positive self-test included anxiety, fear, and shock. One rural RCS feared that the psychological impact of a positive self-test may lead some people to self-harm. Negative responses were expected to be particularly severe among people who had lost a loved one to COVID-19 and those with underlying health conditions.

Reference 2: 0.21% coverage

Hence, there was disagreement whether a positive self-test would lead to any behaviour changes compared with current testing forms. Informants were unclear whether individuals would report their results or self-isolate following a positive self-test.

Reference 3: 0.32% coverage

As with reporting self-test results, informants were unsure whether people would disclose their results to contacts, whether due to fear of stigma or being blamed for another person’s illness or death. Several informants indicated the need for community education campaigns aimed at destigmatising COVID-19 and informing the public about resources available following a positive self-test.

[Files\\Included articles\\Stakeholders perspective of, and experience with contact tracing for COVID-19 in Ghana- A qualitative study among contact tracers, supervisors, and contacts](d64e5f0f-8bc1-4eaa-89c9-12808f4ee8d2)

2 references coded, 0.68% coverage

Reference 1: 0.03% coverage

efusal of some contact to test,

Reference 2: 0.65% coverage

In the view ofsome supervisors and contact tracers, there was deep-seated politicisation ofthe response to COVID-19. Transparency was, therefore, a problem that resulted in dealing with issues oftruth among both contact tracers and contacts. As the cases spread to other regions in the country, the contact tracing was decentralised; however, resources were still managed from Accra. This negatively affected regional and district response to the COVID-19 and contact tracing. The following illustrates these points: “Coordination is a big problem in this entire COVID-19 response. Politicians are at the forefront ofthe fight. Every Ministry tries to do something without a centralised coordination system. So, there is a lack ofharmony in the activities” (S003)

[Files\\'Better to die of disease than die of hunger'- the experience of Igwes (traditional rulers) in the fight against the COVID-19 pandemic in rural South-east Nigeria](4b886fd6-2d7d-4db8-a3f7-e828c98da09c)

1 reference coded, 0.12% coverage

Reference 1: 0.12% coverage

Doubt about the existence of the virus and widespread poverty were found to be the major hindrances in the fight against the pandemic.

[Files\\Included articles\\A qualitative study to explore primary health care practitioners' perceptions and understanding regarding the COVID-19 pandemic in KwaZulu-Nata South Africa](159cbec6-a3f7-40b1-8588-60b5c87fd03e)

5 references coded, 1.94% coverage

Reference 1: 0.12% coverage

Fear The participants used various terms to express a sense of fear related to COVID-19. This fear stemmed from what participants perceived as the negative social media

Reference 2: 0.27% coverage

information and was inherent in the fact that they were unaware of how the disease would eventually manifest if and when it arrived in South Africa. The following statements were made by participants in this regard: ‘It was terrifying in that it included sudden death, and the statistics from other countries as well – so it was frightening then.’ (P01, male, Clinical nurse practitioner)

Reference 3: 0.26% coverage

‘I was so afraid, I thought if this pandemic would come, I became scared for myself and my family, because I know if this thing is airborne, you cannot escape.’ (P04, female, Operational nurse manager) ‘We had already seen from social media how the virus appeared to be killing lots of people so I had a lot of fear and anxiety about what it would do here.’ (P08, male, Clinical associate)

Reference 4: 0.64% coverage

Denial Certain participants believed that the pandemic would be limited to China and would not affect them because China is perceived to be far away from South Africa. Participants also http://www.phcfm.org • Positive community attitudes • Negative attitudes revealed that they did not believe the outbreak was serious because the disease was described as being similar to any normal respiratory illness. This was supported by the following statements: ‘I just did not see it coming, you know. It is just one of those things.’ (P02, female, Social worker) ‘We really did not think it was going become a pandemic. It had not been ruled as a pandemic as of yet.’ (P03, male, Physiotherapist) ‘I did not really think it would occur or spread all over the world because it was said to have originated from a seafood market in China hence, I thought it would stay there so I did not pay much attention to it.’ (P12, female, Professional nurse)

Reference 5: 0.65% coverage

Negative attitudes and behaviours Certain participants revealed that the community had negative ideas about the origin and course of the disease, and negative attitudes stemming from myths and misconceptions about the COVID-19 pandemic. The participants revealed that their perception of negative community attitudes was manifested in blatant refusals to follow the principles of prevention, such as the wearing of face masks and social distancing, which found validation in the following statements: ‘When it first came people did not really want to adhere to wearing masks, such things, and they had their beliefs that this is not for them.’ (P02, female, Social worker) ‘Some patients still seem to not want to accept that COVID-19 [coronavirus disease 2019] is a reality, some think that COVID-19 is Open Access Page 8 of 11 a disease for a specific colour and class of people and not them.’ (P11, female, Enrolled nursing auxiliary)

[Files\\Included articles\\Challenges and opportunities for improved contact tracing in Ghana- experiences from Coronavirus disease-2019-related contact tracing in the Bono region](ba548f54-6213-4aef-bb9c-9acbb476c240)

4 references coded, 2.14% coverage

Reference 1: 0.41% coverage

These include inadequate personal protective equipment, harassment by contacts, politicisation of the discourse around the disease, stigmatization, delays in processing test results, poor remuneration and lack of insurance package, inadequate staffing, difficulty in locating contacts, poor quarantine practices, poor education on COVID-19, language barrier and transportation challenges. Opportunities for improving contact tracing include cooperation, awareness creation, leveraging on knowledge gained in contact tracing, and effective emergency plans for future pandemics.

Reference 2: 0.55% coverage

Politicisation of the discourse around the COVID-19 outbreak The politicisation of the discourse around the disease was another challenge encountered by almost all contact tracers, as it was passionately reported by each group. Some Asare et al. BMC Infectious Diseases (2023) 23:335 Page 5 of 14 contacts requested the political affiliations of contact tracers before accepting to cooperate. This was because they believed that the government had an ulterior motive for how the disease was managed. A discussant in District E remarked that: Politicising the disease is a challenge to us [contact tracers]. This is because you will get to a contact’s home and they start to politicise the entire process [of contact tracing] and they begin to ask you which party you belong to. (GE, male, 43 years old)

Reference 3: 0.77% coverage

Stigmatisation Seventeen discussants, from all six districts, reported stigmatisation as one of the difficulties they encountered during contact tracing. It was revealed that both contact tracers and contacts experience it. It was realised that most contacts were unwilling to provide information about their potential contacts or accepted contact tracers because of possible stigma from society. One participant from District D said: I think stigmatisation is one of the major challenges and is still there. We have a psychologist who does the calling and informs you that you have contact with this person. The moment they here contact with this person, you will trace but will not get them. Sometimes just the fear alone to come out and even give you the details you need is a problem. Because he/she fears that, when he gives out the information and others get to know this person is a contact to the positive case, they will run away from them. There was a situation where everybody was running away from the contact because they thought he has brought the disease. So, stigmatisation is a big challenge. (GD, male, 35 years old)

Reference 4: 0.41% coverage

One major challenge we face, especially when the disease was discovered and spreading to other districts, is stigmatisation. People who had the disease were stigmatised. So, when you are going to trace a contact about a particular case, it is very difficult. People are not willing to own up to the responsibility to say I am a contact to this client. Because they feel if they disclose that, they will also be stigmatised, it is a challenge to us, the contact tracers in identifying who a real contact is. Even some health workers have been ejected from their homes due to stigmatisation. (GE, female, 37 years old)

[Files\\Included articles\\Confronting ‘chaos’- a qualitative study assessing public health officials’ perceptions of the factors affecting Tanzania’s COVID-19 vaccine rollout](1a25f6e5-39c2-4b4e-a4d8-5768224935ca)

1 reference coded, 0.74% coverage

Reference 1: 0.74% coverage

While most respondents praised President Hassan’s government as facilitating vaccination, respondents noted that divergent approaches between Presidents Magufuli and Hassan have engendered confusion and mistrust. For instance, a rural district health secretary noted, ‘the community is still embracing the guidelines of the previous phase, that is why the response towards the issue of vaccination is still low.’ A district health secretary in a different rural district further highlighted ‘the impact of political statements and statements especially in [Magufuli’s] government has made the community distrust the security of the vaccine’. Similarly, a member of an urban HFGC highlighted a ‘lack of information and trust in the reporting authorities’ as pervasive in their community. The project manager of a rural NGO put it even more forcefully:

[Files\\Included articles\\Coping_with_Denialism_How_Street_Level_B](26f35c7b-11ff-47f2-8cd6-03ed4e1113b5)

2 references coded, 0.51% coverage

Reference 1: 0.22% coverage

Fear and stigma presented further difﬁculties. One district health ofﬁ- cial expressed frustration about people “hiding information about patients and . . . locking them in due to fear of stigmatization and isolation by community members.” They explained that hiding patients made it difﬁcult to trace their whereabouts and their contacts.

Reference 2: 0.29% coverage

On the other hand, fear ofCOVID-19 and related psychological distress were mentioned in a number of interviews, particularly as more cases began to be conﬁrmed throughout the country. In some cases, this reﬂected citizens being triggered by memories of recent disease outbreaks. As one district ofﬁcial noted, “All burials that occurred before May 2020 were handled like Ebola related cases and it created fears to members of the general public.”

[Files\\Included articles\\COVID-19 Testing- A Qualitative Study Exploring Enablers and Barriers in the Greater Accra Region, Ghana](c3ce4fa7-5196-43e0-939f-6d855a03ba02)

4 references coded, 2.82% coverage

Reference 1: 1.06% coverage

Sociocultural Perspectives and Practices This study revealed a salient aspect of how a sociocultural perspective of the members of the Ghanaian community inﬂuenced testing and general response to COVID-19. The participants expressed a dilemma as to how people are impacted by Ghanaian culture positively and negatively. The participants mentioned some positive cultural impacts on COVID-19, such as practice of hand hygiene (23). On the other hand, the majority of the study participants mentioned other negative sociocultural inﬂuences on people’s behavior and beliefs toward adherence to COVID-19 protocols, for example, a high dependency on neighbors’ negative personal experiences about COVID19 testing rather than accurate information from the health experts. Thus, people would like to be tested based on shared experiences of their close friends and relatives. A laboratory scientist mentioned, “Ghanaians are particular about maintaining relationships with family and friends. So, when the pandemic occurred, people shared their uncomfortable experience with nasal swabs for testing, and some of them have a fear of getting tested.”

Reference 2: 0.86% coverage

Misconceptions About COVID-19 and Testing Almost all the participants pointed out misconceptions about COVID-19. They emphasized that some people hesitate to get tested because they consider COVID-19 as just common ﬂu. Furthermore, some people believe in rumors that coronavirus does not exist. According to our ﬁndings, these factors inﬂuenced the decision of community members to be tested for COVID-19. “Some people believe in fake news and misinformation that the virus is not real, so vaccination and testing are unnecessary. Also, July 2022 | Volume 10 | Article 908410 Ha et al. Qualitative Study: Factors Inﬂuencing COVID-19 Testing in Ghana they think COVID-19 is just like ﬂu, and big countries are using it for political purposes”—A Community Leader 1. “I have a patient who strongly believes that the virus does not exist. So, I think educating them to adhere to health protocols using TV and radio is essential.”—A Nurse 1

References 3-4: 0.90% coverage

Fear of Stigmatization for Testing Positive Some participants also shared their experiences with patients and community members. According to them, most people were afraid of being isolated and stigmatized by their members of the public, including their workplaces and community. The respondents highlighted the feeling of guilt and shame for people testing positive, which deters others from coming for testing. These ﬁndings support studies showing that healthcare workers, COVID-19 recovered patients, and suspected persons of COVID-19 have faced various forms of COVID-19-related stigma and discrimination, such as stereotyping, social exclusion, mockery, ﬁnger-pointing, and insults in Ghana (24, 25). “If you are diagnosed as COVID-19 positive, people will not even allow you to go to this particular neighborhood; for example, as a COVID-19 worker, people think I will also be infected by the virus one day. So they try not to be close to me.”—Laboratory Scientist 2

[Files\\Included articles\\Diagnostic radiographers’ experience of COVID-19, Gauteng South Africa](3ee8a49c-e68a-4298-a8e2-435fee6c4e53)

4 references coded, 4.26% coverage

Reference 1: 1.58% coverage

New work ﬂow and operations Medical imaging departments' operations were affected by the decrease in imaging referrals at the onset of the lockdown.49 Even though it has been viewed as an opportunity to address “overuse and overdiagnosis”,50 the reduction in imaging referrals, has resulted in some radiographers in the private sector experiencing pay cuts: “…since the lockdown patients were advised not to come to the hospital unnecessarily and with that in private sector, we were affected economically. Less patient ¼ less proﬁt to the company then salaries we cut off” R34 Radiographers' experiences conﬁrm the introduction of new work ﬂow and operations as well as the need to conform to new changes.26e29 The quotes below convey radiographers’ experiences of the changes: “A lot of protocols and precautionary steps taken. Even with all that, people are still confused about what to do, even staff members of other disciplines.” R17 “In my workplace we do not have a set protocol in place for suspected and conﬁrmed cases that are understood by everyone, it sometimes feels like protocols change every day based on who is in charge on that particular day.” R3 “Exhausted from shift changes. Getting irritated with rules that don't make sense.”R59

Reference 2: 0.53% coverage

“Tired ofnot being able to breathe properly because of the mask that I have to wear the whole day. Tired of PPE (I know it's there for my safety but still)…. I often question myself with regards to the PPE. Did I don properly? Did I doff properly? Did I touch my face? Was my hair exposed? R10 “Even though all aspects ofPPE and sanitising is observed, there is still that incessant doubt and fear, just ever lingering” (sic) R57

Reference 3: 0.69% coverage

Social distancing at work was found to be challenging but the support from colleagues was appreciated. Of concern was the lack of clinical history provided by requesting doctors along with the lack of feedback provided to the medical imaging departments for persons under investigation for COVID-19: “…. patients, colleagues & other staff members are in contact with you all the time. It is very difﬁcult to always remind yourself to distance yourself 1,5 m away from people.” R33 “…. patients under investigation, no feedback from the doctors.”

Reference 4: 1.46% coverage

Not being considered frontline workers, a concern echoed by professional bodies impacts allocation of resources as well as radiographers’ well-being:58e64 “It has been rather negative, as we do not get proper personal protective equipment (PPE), we are not recognized as Frontline workers, and we are not considered by our hospital.” R48 “Very nerve wrecking. It's a learning curve so you have to be on guard all the time. Being a radiographer, you are essential but not considered total frontline. With everyone testing the waters it's difﬁcult to have proper guidelines.” R15 There was also concern about the volumes of COVID-19 infor- mation. Information overload coupled with misinformation and conspiracies results in psychological distress.65,66 “At the beginning there was very limited factual information about the virus itself and an overload of information about different theories/conspiracies about covid-19 and this has in turn resulted in so much confusion and difﬁculty to ﬁnd the path of how to deal with the situation and ﬁnd a way forward. There were many arguments and different opinions of how one should go about doing things.”R51

[Files\\Included articles\\Misconceptions, south africa](d853fdac-8274-483c-8330-22c1886f0950)

11 references coded, 4.30% coverage

References 1-2: 0.15% coverage

We found that false information circulated on social media not only instigated confusion, fear and panic, but also contributed to the construction of misconceptions, othering and stigmatizing responses to Covid-19.

References 3-4: 0.14% coverage

The study findings bring attention to the importance of developing communication materials adapted to specific communities to help reduce misconceptions, othering and stigmatization around Covid-19.

Reference 5: 0.44% coverage

False information distributed on social media also caused confusion, fear and panic amongst South African communities. In some instances, healthcare workers reported that the distribution of false information influenced public responses to the screening and testing campaign: “Like as clinic staffwe go in door- to-door, there are incidences where a house owner would refuse for us to go in, saying we don’t want your vaccines because they have Corona. Then we had to explain that we are not injecting people, we are just screening and asking questions. People are really scared, because ofwhat they heard. . .” (Female, community health worker: Western Cape)

Reference 6: 0.67% coverage

Inasmuch as qualitative findings point to study participants having correct knowledge and awareness regarding the transmission and prevention ofCovid-19, myths and misconceptions about the disease was also evident in the data (see Table 4). In the first instance, study participants revealed that young people do not see themselves at risk ofbecoming infected with the virus that causes Covid-19. Hence, they raised concerns that the youth were not taking the Covid-19 pandemic seriously: “My issue is that the younger ones are not taking it seriously. Even though it will mostly kill the elderly since their immune system is compromised, the young and middle aged are not taking it seriously. They think it is one ofthose things that will just pass but the most vulnerable and most at risk are the elderly and the young ones because their immune system is still weak, it is compromised. Those are the people who are often sick. But it is mostly going to affect and kill elderly people” (Male, faith based leader: Gauteng)

Reference 7: 0.22% coverage

At the other end ofthe spectrum, data revealed that there is a discourse ofCovid-19 denialism: “. . .“. . .Firstly it will go back to the issue ofmisconception. You will have people who will come up with their own theories and about [the] virus, you will even get people who says there is no virus” (Female, shebeen patron: Western Cape)

Reference 8: 0.60% coverage

Myths and misconceptions about who is more vulnerable to Covid-19 were based on who was first diagnosed with SARS-CoV-2 in South Africa. Travelers from Italy were the first group to be diagnosed with SARS-CoV-2 in South Africa. Similarly, those considered to be wealthy were more vulnerable to becoming infected with Covid-19, than those from poorer communities: “It [Covid-19] does not affect the poor. It affects the rich because obviously in South Africa it came through a person coming from overseas” (Female, person living with HIV: KwaZulu-Natal) “Those from the township . . . okay, think the virus is for the middle class, upper class and that they cannot get the virus because they do not travel to other countries and are not tourists. They also believe that this virus is for wealthy people, who have money and ‘white’ people, not Africans or ‘blacks’” (Female, person with disabilities: Western Cape)

Reference 9: 0.84% coverage

Misconceptions that people ofa higher socio-economic status were perceived to be more vulnerable to SARS-CoV-2 than others appeared to be common across key informant interviews. Having travelled overseas, race and class were constructed as protective factors against Covid-19. Claims that the disease is a “white-man’s disease”, were influenced by assertions that those assigned ‘black’ in South Africa are naturally immune to the disease. “There is a perception that again it is a ‘white’ person disease because ‘white’ people, and I say that in inverted commas are, the ones who travel overseas and they brought it to Africa. Again my perception is there is a lot ofknowledge and understanding that it’s not about ‘white’ people who also travel ‘black’ people also travel, when we first found out about the first lot ofpatients in KwaZulu-Natal. I know that one ofthe people who has gone overseas was an African woman in my mind are these people not educating themselves enough or do they just block out oftheir mind that this was an African woman who went there. There perhaps it becomes a class thing, lower class people don’t look at it as if they would [because it is] upper class people who travel a lot” (Transgender woman, sexual and gender minority group member: Gauteng)

Reference 10: 0.70% coverage

“At first, I’m [going to] sound racist but they claim that only people who are ‘white’ will be infected by people who are mostly interacting with [‘white’ people]. Plus, that only ‘white’ people can get infected” (Pregnant woman: Gauteng) “Some people used to say that it can’t affect ‘black’ people. They thought it was for ‘white’ people. Ja that was mostly [what was said] out here that it doesn’t affect ‘black’ people, we have strong genes and so on” (Male, community health worker: KwaZulu-Natal) “People are taking it as a joke, it’s lockdown but people are still all over. They only think it will affect the old people with chronic diseases alone, not the young, youth. They don’t want to wash [their] hands, [they] don’t want to cover when one is coughing or sneezing, it’s supposed to be a lifestyle thing not to do it because there is Corona. They also believe it was not meant for Africans, it was for China and Italy and all those stories” (Female, community health worker: Western Cape) Such misconceptions informed othering and stigmatizing responses to Covid-19.

Reference 11: 0.55% coverage

n addition to the stigmatizing responses towards people ofAsian decent with the onset of the Covid-19 pandemic, study participants also made an association between HIV-related stigma and the stigma attached to Covid-19: “There is a stigma attached to it. Let’s keep it to ourselves. No one should know that, you know. Yeah, I think it is the same thing. We reacted [the same way] when people were getting AIDS in our communities” (Male, airport worker: KwaZulu-Natal) “I think obviously discrimination and stigma will be the main thing. Because I will make an example ofHIV in the block ofthe flats that I live in. People are still. . .very discriminatory against people who are living with HIV. So with Coronavirus you know I am sure that person will be excluded from everything. . .” (Female, person living with HIV, KwaZulu-Natal)

[Files\\Included articles\\Preparedness and response to covid-19 in Woreta Town, North West Ethiopia](bf44178e-777e-489a-ba3d-c59766ae3cd6)

5 references coded, 1.60% coverage

Reference 1: 0.79% coverage

Risk communication and community engagement Awareness creation and communication activities were done inside and outside the health center using different mech- anisms such as; home visits, leaﬂets, loud speaker-loaded cars, inﬂuential persons (religious leaders and political leaders), scheduled teachings arranged for patients and their care givers. In addition, public shows were made in which health workers have travelled in procession along the main street to transmit COVID-19 prevention message. Similarly, a mask-wearing show was held by governmental workers including the police, the health professionals and other civil servants. Apart from the above, people were getting information by their own effort from national TV, radio, telecom voices, or from social media accounts. However, the communication tasks were less fruitful since misunderstandings, groundless rumors, suspicions and denials are still prevalent. According to kebele 01 and 03 administrators, the main challenge for prevention was the politicized outlook and negligence against the government’s Covid-19 response activities.

References 2-3: 0.40% coverage

Denial and suspicion also existed in some members who got diﬃculty in accepting the health professionals’ expression about the disease. For instance, an individual who was tested positive and sent to isolation center has the following comments. “Security forces and health professionals have suddenly come with their car around the bus station and ordered me to go to an isolation center in another town. They did not allow him even to arrange things. I stayed there for 19 days. I was healthy, I don’t know why they referred me to an isolation center”, a resident from kebele 03

Reference 4: 0.14% coverage

“I was healthy, I don’t know why they referred me to an isolation center”, a resident from kebele 03 “Sadly, some people tag us ‘corona’ as if we have brought it to them”. A health extension worker in kebele 01

Reference 5: 0.27% coverage

and residents were seriously following this case. Parallel to this development, there was a rumor that laboratory results of Covid-19 cases were consciously fabricated to turn the public attention away from the hot political issues. Since the rumor was quickly disseminated, some people dared to deny its existence while others concluded that Covid-19 was not a serious health threat.

[Files\\Included articles\\Self testing for COVID 19 in Durban and Eastern Cape South Africa a qualitative inquiry targeting decision takers](6ca4769f-dd80-4c55-b8ad-484b514bf85b)

18 references coded, 5.28% coverage

Reference 1: 0.07% coverage

Generally, rural informants described a high degree of trust in public-sector testing.

Reference 2: 0.31% coverage

The barriers identiﬁed to conventional facility-based testing were diverse. The expense of COVID-19 testing was mentioned by all groups, either incurred from lost wages while waiting in queues for government testing or the cost of private testing. The cost of transportation was a barrier in King Sabata Dalindyebo, where testing facilities are geographically dispersed.

Reference 3: 0.14% coverage

Broadly, stigma and fear were the most cited social barriers to testing. Stigma was associated with the fear of being seen testing (particularly in King Sabata Dalindyebo),

Reference 4: 0.06% coverage

of a positive result, and of disclosing positive results to contacts.

Reference 5: 0.33% coverage

Fears of being separated from family, losing income, and isolation were also cited. One element, called an ‘ostrich mentality’ by a female PI, aﬀecting willingness to seek testing was people’s tendency to prefer not to know whether they have acquired SARS-CoV-2 to avoid the hassle of requisite actions. As this female PI put it: ‘If you don’t know [that you have SARS-CoV-2], you don’t have to deal with it.’

Reference 6: 0.27% coverage

Barriers to access testing were drawn along socio-economic and education lines. In the rural area, informants described how COVID-19 was once considered a disease of ‘people who have money’, which has contributed to ‘ﬁnger-pointing’ toward the SARS-CoV-2 car- riers and the persons who develop symptomatic COVID-19 disease.

Reference 7: 0.51% coverage

Potential disadvantages were related to concerns about the potential lack of account- ability aﬀorded to people who would use self-testing and whether people would disclose their results, seek appropriate care, or self-isolate at the risk of lost income or social ostracisation. There were worries that widespread use of self-testing would compromise reporting and surveillance, should people be unable to report their results. I’m not sure if they’ll give their details to surveillance mechanisms wherever they are…So if people do their own tests, we may not know who has COVID and what the numbers are. (PI, 51, female)

Reference 8: 0.56% coverage

It was felt that individuals who would be less likely to use self-testing are those who are ‘complacent’ about COVID-19, those who ‘passively accept’ the situation, and those who deny the COVID-19 pandemic. While it may appear that this contradicts views of selftesting as ‘liberatory’, this is not the case. For those who are described as ‘unconcerned’ or ‘complacent’ about the COVID-19 pandemic and its impact, self-testing has little relevance since they are thought to be unlikely to test at any rate. Nevertheless, a few informants opined that access to self-testing may be a mechanism for increasing the reluctant persons’ uptake of SARS-CoV-2 testing if symptoms are evident.

Reference 9: 0.91% coverage

The trustworthiness of the distributor was an important consideration to facilitate uptake. Compared with Durban institutions, rural healthcare institutions were considered more trustworthy for distributing self-testing. One PI, a male public service administrator, felt that NGOs are not trustworthy. An RCS, the female head of an academic department, stated that people in Durban would not trust door-to-door initiatives delivering selftesting. In contrast, many rural RCSs felt that religious leaders and chiefs would have suﬃcient rapport with the population to encourage follow-up activities by self-test users. Many informants referred to local community leaders as the appropriate individuals to provide education about and even administer self-testing. I think people do trust their pastors, their healthcare workers, nurses and general practitioners, pharmacists, principles…They generally don’t trust politicians. So, I wouldn’t include them there. But, generally, the community leaders, non-politically aligned, I think would be people that would be trustworthy. (RCS, 60, male)

Reference 10: 0.41% coverage

The possible harms caused by a positive self-test were considered to mirror the harms that could follow a positive provider-initiated test. Potential negative consequences of a positive self-test included anxiety, fear, and shock. One rural RCS feared that the psychological impact of a positive self-test may lead some people to self-harm. Negative responses were expected to be particularly severe among people who had lost a loved one to COVID-19 and those with underlying health conditions.

Reference 11: 0.06% coverage

Social stigma resulting from a COVID-19 diagnosis was also described.

Reference 12: 0.14% coverage

Beyond the psychological impacts, informants identiﬁed signiﬁcant economic consequences of a positive self-test, in the form of lost income or dismissal from one’s job

Reference 13: 0.11% coverage

There was agreement that the public currently struggles to disclose their test results and self-isolate with traditional testing.

Reference 14: 0.15% coverage

Several RCSs and PIs expressed frustration that shortcomings in the healthcare system may render the communication of positive self-test results to health authorities challenging.

Reference 15: 0.20% coverage

HCWs tended to feel that ‘responsible’ people would report their results and disclose them to their contacts, but this would also be dependent on the perceived consequences a positive self-test would have for people’s ﬁnances and employment.

Reference 16: 0.63% coverage

They also felt that symptomatic individuals would be more likely to report. A male, rural physician described how the actions required for a positive self-test may compromise an individual’s freedom and how fear of this consequence could deter people from reporting. In contrast, a rural, male, traditional leader felt that people would readily communicate with clinics because they have experience communicating with healthcare providers for other diseases. People don’t like any of their freedom to be taken away from them. People feel that if they have to isolate, their freedoms are being taken from them…They don’t want that information to be spread to the general population of the clinic because it may cause people to ask of them to stay at home…(HCW, 37, male)

Reference 17: 0.32% coverage

As with reporting self-test results, informants were unsure whether people would disclose their results to contacts, whether due to fear of stigma or being blamed for another person’s illness or death. Several informants indicated the need for community education campaigns aimed at destigmatising COVID-19 and informing the public about resources available following a positive self-test.

Reference 18: 0.12% coverage

Informants felt that individuals’ willingness to isolate would be determined by whether they would lose income should they take sick leave.

[Files\\Included articles\\Stakeholders perspective of, and experience with contact tracing for COVID-19 in Ghana- A qualitative study among contact tracers, supervisors, and contacts](d64e5f0f-8bc1-4eaa-89c9-12808f4ee8d2)

7 references coded, 3.11% coverage

Reference 1: 0.10% coverage

OVID-19 related stigma also served as a motivator for self-quarantine. One stakeholder shared his views as follows:

Reference 2: 0.21% coverage

“You know it is a new disease, and people who are infected are stigmatized. So people selfquarantined to stay away from their community members so they would not be identified or frequently seen in public after having been tested positive” (CT005)

Reference 3: 0.29% coverage

Another contact revealed that her employers assured her ofreceiving all benefits due to her whilst in quarantine. Hence, it was easy to comply as indicated: “My employers sent a well-wishes message to me. They told me that all my salary and allowances would be paid even though I have not been coming to work because ofthe situation I find myself” (C003)

Reference 4: 1.15% coverage

At the time ofthe study, the death toll among countries in Europe, America, and Asia that were first affected by the pandemic was so high, and many had anticipated it to be worse in Africa. This was mentioned as a reason for compliance with the directive to self-quarantine. “You see how people are dying in developed countries and the situation is likely to be worse in Africa. So I have to self-quarantine to prevent my family and close relatives from getting infected in case I am tested positive” (C004) Contacts experience with the health workers during contact tracing Contacts revealed that they were initially afraid and felt uncomfortable when they were told they had to self-quarantine and screen for COVID-19. This directive, according to participants, was informed by the high level ofCOVID-19 related mortality that had been reported across the world. They indicated that the most troubling part was having to stay in your room and not interact with your family. This, according to contacts in this study, saddened their hearts and left them thinking for several days. “I was initially very anxious when I was told I was a contact and had to be screened for the condition. It is not an easy experience, especially the way people were dying from the condition outside the country. I had to also live in my room alone, and food is virtually left at the door for me” (C002)

Reference 5: 0.52% coverage

Nonetheless, some contacts indicated they did not like the daily visit because ofthe stigma. As a result, some contacts indicated they had to procure their own thermometer to prevent the daily visit by the contact tracers. One ofthem stated as follows: “They were coming every day, but I complained. I told them not to come because there is a stigma. Ifyou sell something, people will not buy it. So, I bought my own thermometer and asked them to call for me to provide them with the information. You know, people in the community would be wondering why health workers come to your house everyday. . . the stigma is there” (C005).

Reference 6: 0.47% coverage

Some contacts also refuse to pick the calls ofthe contact tracers. This was, therefore, in a way, undermining the process. A supervisor shared the following in an interview: “Sometimes your contact tracer come and tell you how some contacts have insulted them. There are lots ofpsychological dimension to it . . .so I encourage the contact tracers not to give up. Some people believe they have low risk so when you list them and want to monitor them, they relocate, or you call them, and they will refuse to pick, and when you go to the house, you don’t find them” (S006).

Reference 7: 0.37% coverage

nevertheless, concerns were raised about daily home visits. Although the study revealed that the communities and contacts were willing to support contact tracers, concerns were raised about stigmatisation ofcontacts. Thus, some contacts felt it was inappropriate for contact tracers to pay daily visits. This, in their view, could accentuate COVID-19 related stigma in the community. This is an important observation in this study.

[Files\\Included articles\\Implementation of a modified drive-through sampling strategy for SARS-CoV-2-the Nigerian experience](5a4904fa-bf54-46ed-8954-5f5dfa243c8b)

2 references coded, 1.93% coverage

References 1-2: 1.93% coverage

Symptoms, waiting phase and the testing experience: all the respondents stated that they had experienced symptoms like cough, shortness of breath, fever, and that they had travel history and had exposure to people who were exposed to confirmed cases. Three of the respondents experienced some form of psychological distress, they were scared to take the test, they had cried and two of them found the test very invasive and painful. Suggestions and Recommendations: overall, knowledge and awareness of COVID-19 are still low and there is a need to educate people, provide counsel, and increase the uptake of the testing. The majority of the clients stated that the test was scary and invasive however, the process and turnaround time was effective and prompt. They suggested that the following strategies should be explored: 1) The test is invasive and painful and other alternatives of testing should be explored. 2) Knowledge and awareness of COVID-19 is low. NIMR should The Pan African Medical Journal. 2020;35 (Supp 2):107 | Olufemi Samuel Amoo et al. 3 educate people and create awareness at different levels. 3) Counselling should be done to reduce anxiety and distress. 4) Few people know about the centre in NIMR, awareness could increase the number of persons tested.

[Files\\Included articles\\Self testing for COVID 19 in Durban and Eastern Cape South Africa a qualitative inquiry targeting decision takers](6ca4769f-dd80-4c55-b8ad-484b514bf85b)

9 references coded, 4.34% coverage

Reference 1: 0.61% coverage

Knowledge of conventional COVID-19 testing Informants’ descriptions of currently available testing services varied. HCWs, urban RCSs, and urban PIs tended to describe testing services in greater detail and were more likely than informants from rural areas to identify antigen, antibody, and PCR tests as being available. Urban informants identiﬁed centralised testing at hospitals, clinics, and community health centres. PIs also identiﬁed private pharmacies as testing sites. Urban informants further identiﬁed ‘pop up’ (temporary, community-facing testing sites) and drive-through laboratories. Only one rural RCS, the leader of a taxi association, gave drive-through and mobile outreach as options in King Sabata Dalindyebo.

Reference 2: 0.58% coverage

In Durban, informants identiﬁed a public–private sector dichotomy. Particularly among RCSs, testing services were described as distributed along a gradient of access, convenience, and cost, with free testing available at government facilities but with long waiting times. In contrast, private testing was considered by RCSs more available but also less convenient due to its cost. Accessing testing was considered burdensome and ‘dangerous’, given fears that SARS-CoV-2 could be transmitted while waiting in queues. In Durban…the only options available are to go to a test center, either to government test centers, which are free and slow, or go to private ones, which are fast and expensive. (RCS, 55, male)

Reference 3: 0.32% coverage

Before, there were all these restrictions on when you could go, and where you could go, and now there’s sort of drive-through concept, and people were nervous about being infected and infecting. So, it became quite complicated. I think people who have access to private healthcare were more able to access testing than people who were more reliant on public healthcare system. (PI, 52, male)

Reference 4: 0.50% coverage

Despite these concerns, generally, informants felt that self-testing would provide numerous advantages, including reduced burden on healthcare workers if individuals could selftest without their assistance. HCWs felt that self-testing would help conserve diagnostic resources. One RCS, a process engineer, felt that self-testing would reduce bureaucratic CONTEMPORARY SOCIAL SCIENCE 457 ‘red tape’ involved in reporting. Should self-testing be provided for a charge, HCWs and PIs also felt that its scale-up may help to reduce the ﬁnancial burden on the health system caused by the high demand of testing.

Reference 5: 0.91% coverage

Self-testing was understood to be a tool that promotes privacy, the lack of which was considered, particularly by rural informants, a deterrent to facility-based testing. Self-testing was considered by RCSs to promote health decision-making autonomy, much like ‘knowing your status’ with HIV/AIDS. Self-testing was viewed as particularly critical for hard-to-reach groups who are unwilling or unable to seek health services. For these groups, such as ‘homeless’ individuals or ‘people who use drugs’, self-testing was seen as in some way ‘liberating’. It meant that these hard-to-reach groups would not have to navigate institutional arrangements, which are often stigmatising and humiliating. Selftesting, then, needs to be grounded within contexts of systemic inequality and selfresponsibilisation governance strategies. Especially with the population that I work with [people who use drugs and homeless people], I think that [self-testing] will allow them the autonomy to do it wherever they’re based and not being forced to go into a facility where they [can be] discriminated. (PI, 31, female)

Reference 6: 0.94% coverage

nformants felt that self-testing had the potential to be more acceptable as a SARS-CoV-2 diagnosis, if it were low-cost, widely available, and had shorter turn-around times for results, compared with provider-performed testing. The psycho-social beneﬁts were also considered an advantage, as self-testing would allow individuals to mentally prepare for testing. Other beneﬁts were the reduced risk of SARS-CoV-2 transmission in crowded facilities and an increased likelihood that individuals could self-isolate quickly. Potential disadvantages were related to concerns about the potential lack of account- ability aﬀorded to people who would use self-testing and whether people would disclose their results, seek appropriate care, or self-isolate at the risk of lost income or social ostracisation. There were worries that widespread use of self-testing would compromise reporting and surveillance, should people be unable to report their results. I’m not sure if they’ll give their details to surveillance mechanisms wherever they are…So if people do their own tests, we may not know who has COVID and what the numbers are. (PI, 51, female)

Reference 7: 0.12% coverage

One rural-based physician felt that poor knowledge about COVID-19 in general and the use of selftesting could lead to the incorrect use of self-tests.

Reference 8: 0.23% coverage

Saliva samples were the preferred sample for self-testing, although HCWs felt that throat or nose swabs would produce the most accurate results. However, the swabbing process was thought unpleasant, and it was suggested that self-testers might collect swab samples incorrectly.

Reference 9: 0.13% coverage

It was felt that most people would want to self-test at home. HCWs indicated this would be essential to reduce the inﬂux of patients to healthcare facilities

[Files\\Included articles\\Implementation of a modified drive-through sampling strategy for SARS-CoV-2-the Nigerian experience](5a4904fa-bf54-46ed-8954-5f5dfa243c8b)

2 references coded, 1.93% coverage

Reference 1: 0.66% coverage

Symptoms, waiting phase and the testing experience: all the respondents stated that they had experienced symptoms like cough, shortness of breath, fever, and that they had travel history and had exposure to people who were exposed to confirmed cases. Three of the respondents experienced some form of psychological distress, they were scared to take the test, they had cried and two of them found the test very invasive and painful.

Reference 2: 1.28% coverage

Suggestions and Recommendations: overall, knowledge and awareness of COVID-19 are still low and there is a need to educate people, provide counsel, and increase the uptake of the testing. The majority of the clients stated that the test was scary and invasive however, the process and turnaround time was effective and prompt. They suggested that the following strategies should be explored: 1) The test is invasive and painful and other alternatives of testing should be explored. 2) Knowledge and awareness of COVID-19 is low. NIMR should The Pan African Medical Journal. 2020;35 (Supp 2):107 | Olufemi Samuel Amoo et al. 3 educate people and create awareness at different levels. 3) Counselling should be done to reduce anxiety and distress. 4) Few people know about the centre in NIMR, awareness could increase the number of persons tested.

[Files\\Included articles\\Self testing for COVID 19 in Durban and Eastern Cape South Africa a qualitative inquiry targeting decision takers](6ca4769f-dd80-4c55-b8ad-484b514bf85b)

12 references coded, 2.84% coverage

References 1-3: 0.50% coverage

Despite these concerns, generally, informants felt that self-testing would provide numerous advantages, including reduced burden on healthcare workers if individuals could selftest without their assistance. HCWs felt that self-testing would help conserve diagnostic resources. One RCS, a process engineer, felt that self-testing would reduce bureaucratic CONTEMPORARY SOCIAL SCIENCE 457 ‘red tape’ involved in reporting. Should self-testing be provided for a charge, HCWs and PIs also felt that its scale-up may help to reduce the ﬁnancial burden on the health system caused by the high demand of testing.

Reference 4: 0.91% coverage

Self-testing was understood to be a tool that promotes privacy, the lack of which was considered, particularly by rural informants, a deterrent to facility-based testing. Self-testing was considered by RCSs to promote health decision-making autonomy, much like ‘knowing your status’ with HIV/AIDS. Self-testing was viewed as particularly critical for hard-to-reach groups who are unwilling or unable to seek health services. For these groups, such as ‘homeless’ individuals or ‘people who use drugs’, self-testing was seen as in some way ‘liberating’. It meant that these hard-to-reach groups would not have to navigate institutional arrangements, which are often stigmatising and humiliating. Selftesting, then, needs to be grounded within contexts of systemic inequality and selfresponsibilisation governance strategies. Especially with the population that I work with [people who use drugs and homeless people], I think that [self-testing] will allow them the autonomy to do it wherever they’re based and not being forced to go into a facility where they [can be] discriminated. (PI, 31, female)

Reference 5: 0.19% coverage

nformants felt that self-testing had the potential to be more acceptable as a SARS-CoV-2 diagnosis, if it were low-cost, widely available, and had shorter turn-around times for results, compared with provider-performed testing.

Reference 6: 0.11% coverage

The psycho-social beneﬁts were also considered an advantage, as self-testing would allow individuals to mentally prepare for testing.

Reference 7: 0.13% coverage

Other beneﬁts were the reduced risk of SARS-CoV-2 transmission in crowded facilities and an increased likelihood that individuals could self-isolate quickly.

Reference 8: 0.51% coverage

Potential disadvantages were related to concerns about the potential lack of account- ability aﬀorded to people who would use self-testing and whether people would disclose their results, seek appropriate care, or self-isolate at the risk of lost income or social ostracisation. There were worries that widespread use of self-testing would compromise reporting and surveillance, should people be unable to report their results. I’m not sure if they’ll give their details to surveillance mechanisms wherever they are…So if people do their own tests, we may not know who has COVID and what the numbers are. (PI, 51, female)

Reference 9: 0.12% coverage

One rural-based physician felt that poor knowledge about COVID-19 in general and the use of selftesting could lead to the incorrect use of self-tests.

Reference 10: 0.23% coverage

Saliva samples were the preferred sample for self-testing, although HCWs felt that throat or nose swabs would produce the most accurate results. However, the swabbing process was thought unpleasant, and it was suggested that self-testers might collect swab samples incorrectly.

References 11-12: 0.13% coverage

It was felt that most people would want to self-test at home. HCWs indicated this would be essential to reduce the inﬂux of patients to healthcare facilities

[Files\\'Better to die of disease than die of hunger'- the experience of Igwes (traditional rulers) in the fight against the COVID-19 pandemic in rural South-east Nigeria](4b886fd6-2d7d-4db8-a3f7-e828c98da09c)

1 reference coded, 0.14% coverage

Reference 1: 0.14% coverage

Results: Findings showed that the traditional rulers adopted measures such as the use of town criers to raise awareness among rural people about COVID-19.

[Files\\Included articles\\A qualitative study to explore primary health care practitioners' perceptions and understanding regarding the COVID-19 pandemic in KwaZulu-Nata South Africa](159cbec6-a3f7-40b1-8588-60b5c87fd03e)

3 references coded, 1.14% coverage

References 1-2: 1.00% coverage

Poor preparation by the Department of Health Participants in this study described an array of experiences, which indicated that they perceived the Department of Health to have been underprepared in responding to the pandemic in South Africa. The perceived poor preparation reported by the healthcare workers in this study was also because of the lack of basic essential resources required to enable clinicians to both care for clients and protect themselves whilst providing care to patients. The following statements support this sub-category of description: ‘There was nothing at all. As a result, we were supposed to be prepared by all PPE [personal protective equipment] gear, but there was nothing at all. Even the PPE that we were using like masks, it was not surgical masks. It was paper masks, which were very poor.’ (P04, female, Operational nurse manager) Open Access Page 7 of 11 ‘When it comes to planning after seeing that something is happening in China and it is spreading to other countries, there was no planning to prepare for when it comes to South Africa.’ (P06, female, Pharmacy manager) ‘I think the government was very late in terms of banning international travel to those countries that were affected. They also responded late in terms of screening and isolation of suspected cases which mean they might have not been adequately prepared despite having verbalised being ready.’ (P09, female, Community care giver)

Reference 3: 0.14% coverage

‘They should have had a way of screening people that are entering the country which is better than the one that they started using which focused on high body temperature only.’ (P07, male, Professional nurse)

[Files\\Included articles\\Challenges and opportunities for improved contact tracing in Ghana- experiences from Coronavirus disease-2019-related contact tracing in the Bono region](ba548f54-6213-4aef-bb9c-9acbb476c240)

12 references coded, 6.35% coverage

Reference 1: 0.68% coverage

Challenges faced by contact tracers I. Inadequate personal protective equipment (PPEs) Inadequate PPEs was one of the major challenges reported by all the participants. All of them agreed that although they were provided with some PPEs, they were inadequate. Gloves and nose masks were the only PPEs supplied to contact tracers. Sometimes, these PPEs were insufficient to effectively carry out their duties on a daily basis. Thus, the contact tracers, on some occasions, would have to purchase personal gloves and nose masks for their safety. A participant remarked that: We have a challenge with PPEs such as the [nose] mask. They are not enough. So, we have to buy it ourselves. Although the situation has improved, they always prioritised those in the intensive care unit so we do not have access to those ones. And the gloves are a bit of a challenge, we do not have enough to use, so mostly we have to rely on our pocket money to buy some of the gloves in handling the samples. (GC, male, 29 years old)

Reference 2: 0.56% coverage

Delays in processing test results Delays in receiving test results were another challenge that affected contact tracing. Twenty-two of the discussants complained that delays in processing test results as a result of inadequate testing centres made it challenging for them to continue the quarantine process. One of the discussants from district A explained that: The testing centre is a challenge to us as a district. We have sent about 27 samples, and it is almost one and a half months now the results are not in yet. One of them was sick, but we don’t know the status of that person till now. Although we have testing centres here, they are doing internal testing, so we have to send our samples to Sunyani [the capital of the Bono region] before they are sent to the central laboratory, which delays a lot. (GA, male, 33 years old)

Reference 3: 0.35% coverage

Another participant from District D reported similar complaints about the delays in processing the testing results. The situation, on some occasions, led to misunderstandings between the contacts and the contact tracers. He said: Sometimes, you can send samples for testing but the results will not come early. This makes the contacts start complaining, especially when they have no symptoms. They will tell you they don’t have any disease which is why you cannot tell them their results. (GD, female, 41 years old)

Reference 4: 1.27% coverage

Poor remuneration and lack of insurance package for contact tracers Poor remuneration and lack of insurance packages for contact tracers were other challenges highlighted by Another participant explained how possibly exposed individuals to the disease hid because of the fear of stigmatisation. She explained that: One major challenge we face, especially when the disease was discovered and spreading to other districts, is stigmatisation. People who had the disease were stigmatised. So, when you are going to trace a contact about a particular case, it is very difficult. People are not willing to own up to the responsibility to say I am a contact to this client. Because they feel if they disclose that, they will also be stigmatised, it is a challenge to us, the contact tracers in identifying who a real contact is. Even some health workers have been ejected from their homes due to stigmatisation. (GE, female, 37 years old) Asare et al. BMC Infectious Diseases (2023) 23:335 Page 6 of 14 almost all the discussants. A discussant revealed that the government had promised a 50% allowance to all frontline health workers and an insurance package for those who had contracted the disease in their line of duty. However, some health workers were yet to receive them as at the time of the data collection. Most of the participants became emotional during a discussion on remuneration. A participant recounted that: The government promised to give a 50% allowance to all frontline health workers; as we speak, some of us have not benefited from it. We don’t know whether the government selected its contact tracers. I am sure our names were submitted but out of the names submitted, they chose what they wanted and paid them, and we were side-lined. Although I still do the work I have something in mind. I will do the work but will not dedicate all my time. (GB, male, 35 years old)

Reference 5: 0.43% coverage

A similar sentiment was shared during one of the discussions. The problem of poor remuneration had worsened to the extent that some contact tracers threatened to quit working. A discussant had this to report: People who were much involved in COVID-19 did not receive the 50% at all. We were here and people who were not involved rather had it. Whiles those who are more involved don’t get the 50% and is a form of demotivation. If Ghana is going to record more cases in which more contact tracing is going to be done, then they need to solve this issue. If not, they should be sure that nobody will volunteer to be a contact tracer. (GE, female, 37 years)

Reference 6: 0.40% coverage

Inadequate staffing According to some nineteen discussants, the increasing number of cases led to an increased workload on contact tracers since the staff strength remained the same. A discussant reported that: It is tedious and a bit challenging because cases are increasing, the number of contacts has to also increase, and you have to trace every day. So, I will say the workload is too much. And also, sometimes you will go to the house but won’t meet the contact, they have already left to the farm and you have to go there several times before you will meet them. (GF, male, 45 years old)

Reference 7: 0.10% coverage

Another participant also explained how workload increased at the peak of COVID-19. He narrated: It got to a time when the workload was very high,

Reference 8: 0.16% coverage

especially at the peak of the outbreak when we had more cases. This gave the extra duty to us [contact tracers] and sometimes you need to go beyond office hours to carry out contact tracing and it was a challenge. (GC, male, 36 years old)

Reference 9: 0.80% coverage

Difficulty in locating contacts A total of twenty-four discussants, from all six districts, reported difficulties in locating contacts. They complained that some of the contacts gave false information and inaccurate contact information. A participant recounted that: One thing we have generally in our country is a poor address system, although you will be given the address of the contact, you are unable to trace it because the street name is not clear or correct. And you will find it difficult in locating that particular person. Sometimes you will be given a particular number to call, and you will try calling but due to a bad network, you will not reach the person. It makes it difficult in locating contact as well. (GD, male, 36 years old) Another participant expressed similar concern by saying: Locating a contact is a bit more difficult here than that in a rural area. Sometimes you can be giving the address to the house but because the house is not numbered you will find it difficult to locate it. You have to call the contact before they will direct you to the house but if you are not lucky and they don’t pick up your call, then it becomes very difficult. (GA, male, 34 years old)

Reference 10: 0.43% coverage

Transportation challenges Despite not being reported as a major challenge, issues related to transportation were reported by three participants from District B. One of them said: The means of transport is a challenge to us, as at when we receive test results from the testing centre, we are supposed to rush into the community. Sometimes there might be a vehicle but no fuel so you have to go by motorbike to the community, which is not supposed to be so. We are not supposed to use a motorbike to convene the sample to the testing site from the household. But the district is having only one vehicle which is sometimes busy. (GB, male, 40 years old)

Reference 11: 0.61% coverage

Leveraging cooperation from community leaders Twelve of the participants revealed that the communities, with the support of their leaders, were cooperative and receptive. Hence, strengthening partnerships in such communities could help ease some of the initial challenges that contact tracers face. A discussant explained: The opportunities were great. For the health sector, everybody was involved from the DHD to the subdistrict level because more awareness creation was done. The community leadership was also involved and we were also fortunate to have a minister coming from here, so the people wanted to cooperate so that his name would not be put in the mud. If we Asare et al. BMC Infectious Diseases (2023) 23:335 Page 8 of 14 can build on these structures going forward and not only wait for another pandemic before we go for their help, I think it will help us a lot. (GA, female, 39 years old)

Reference 12: 0.56% coverage

Effective emergency preparedness on the part of health authorities Almost all the groups noted that the preparation by Ghana Health Service (GHS) for the fight against COVID-19 was inadequate. This was revealed in the challenges encountered with the supply and distribution of PPEs and personnel. They believed that the GHS should learn from these experiences and prepare adequately for future pandemics. One discussant explained: Asare et al. BMC Infectious Diseases (2023) 23:335 Page 9 of 14 When COVID-19 actually came, we [the Ghana Health Service] were not prepared, which is why we faced a lot of challenges with contact tracing in the beginning. The PPEs were not there, yet we had to work. So, if the authorities could learn their lessons, I think we will be better prepared for the future. (GE, male, 36 years old)

[Files\\Included articles\\Confronting ‘chaos’- a qualitative study assessing public health officials’ perceptions of the factors affecting Tanzania’s COVID-19 vaccine rollout](1a25f6e5-39c2-4b4e-a4d8-5768224935ca)

1 reference coded, 0.99% coverage

Reference 1: 0.99% coverage

Another issue was lack of testing capacity for COVID- 19. An urban health secretary stated: ‘we don’t have a laboratory that can detect COVID- 19, so the health provider 6 only measures temperature, heart rate and blood pressure…so they may vaccinate a person who is infected.’ Several respondents mentioned this challenge, saying they knew people who died after receiving the vaccine, suggesting that the deceased were likely infected prior to vaccination. A common concern among respondents was lack of training for medical providers. A rural village officer stated that they lacked ‘experts to provide education on this disease’. An urban ward officer explained that no training was given to them on COVID- 19 nor the vaccines and their side effects, making it difficult to answer the public’s questions. Officers in another urban area reported receiving what they described as unofficial training on COVID- 19 organised by WHO and the government, on ‘immunization and vaccine storage’. Respondents reported that inadequate training led to healthcare providers’ misconceptions, including whether or not pregnant and breastfeeding women can be vaccinated.

[Files\\Included articles\\Coping_with_Denialism_How_Street_Level_B](26f35c7b-11ff-47f2-8cd6-03ed4e1113b5)

6 references coded, 2.51% coverage

Reference 1: 0.56% coverage

Surveillance In March 2020 the MoHCDGEC issued a series of standard operating procedures (SOPs) that included surveillance for COVID-19. The guidelines stated that all patients with acute respiratory illness should be screened for COVID-19 and that health care facilities, especially emergency centers and outpatient clinics, should identify an appropriate area and person to do the screening (MoHCDGEC 2020). Health facilities were advised to use a national screening checklist, which included symptoms, travel history, and contact with probable or conﬁrmed cases. Travelers, especially those from countries with COVID-19 outbreaks, were to be quarantined for 14 days and tested. However, it is important to note that on May 3, 2020, President Magufuli suspended the head of the national laboratory, just before stopping all COVID-19 surveillance

Reference 2: 0.22% coverage

Regional and district ofﬁcers were primarily responsible for coordinat- ing testing but reported insufﬁcient laboratory capacity. They complained that the national laboratory experienced substantial delays in processing coronavirus tests. District health ofﬁcers said that the laboratory took one to two weeks to deliver results.

Reference 3: 0.20% coverage

One respondent mentioned that sometimes the laboratory results seemed inaccurate in that “a patient tested negativewhile he/she had critical signs.” Respondents across levels also reported difﬁculties testing and quarantining travelers because of a lack of resources for maintaining contact with travelers.

Reference 4: 0.05% coverage

The primary challenges were insufﬁcient laboratory capacity for testing

Reference 5: 0.89% coverage

Surveillance Respondents reported that they decentralized surveillance by involving ward/village leaders and community health workers in the surveillance process. Regional ofﬁcers trained community health workers to help with COVID-19 surveillance. Several district ofﬁcers reported increasing their capacity by mobilizing existing community health workers and training them to conduct surveillance. They did this without a speciﬁc mandate to do so in the central SOP (MoHCDGEC 2020). One DMO reported their innovative approach to surveillance that reached across government levels: “We created a WhatsApp [group] with the DMO, surveillance ofﬁce, ward and village leaders for timely reporting and response. For example, when there is a new visitor in a village or a suspected case, it is shared on the WhatsApp group and the respective team acts in a timely manner.” Ward ofﬁcers also helped with surveillance. They “followed up at the street level to observe any suspected corona cases ...incollaboration with health workers.” They also reported to ward executive ofﬁcers any guests/travelers who were in their areas, as this village executive ofﬁcer explains: “We gave information about corona suspects within our street. We reported about guests in our streets from outside the country or nearby regions to ward executive ofﬁcers ...the security ofﬁcials.”

Reference 6: 0.60% coverage

Communication Respondents unanimously agreed that their public education activities were their most effective adaptations. Although the MoHCDGEC-issued SOP mentionedcommunity education as important for epidemic response, it did not detail speciﬁc strategies(MoHCDGEC2020). Our respondents felt that collaboration with community leaders was critical for delivering effective public health education. Respondents described transmitting COVID-19 Tanzania COVID-19 Lessons 1005 Downloaded from http://read.dukeupress.edu/jhppl/article-pdf/46/6/989/1382580/989carlitz.pdf by guest on 17 November 2021 1006 Journal of Health Politics, Policy and Law prevention information via leaﬂets and posters, mobile phones, local radio, public announcements (loudspeakers), and songs. Interviewees also stressed the importance of reaching places such as motorcycle depots, markets, and places of worship

[Files\\Included articles\\COVID-19 Testing- A Qualitative Study Exploring Enablers and Barriers in the Greater Accra Region, Ghana](c3ce4fa7-5196-43e0-939f-6d855a03ba02)

21 references coded, 12.49% coverage

References 1-2: 0.21% coverage

Better health governance through political leadership, community participation, multisectoral collaboration, effective resource management, and information systems played a crucial role in catalyzing COVID-19 testing.

References 3-4: 0.29% coverage

The primary barriers to testing were mainly COVID-19 infodemic, inadequacy of material resources to meet growing health needs, and a lack of opportunities to have equal and easy access to testing services. Furthermore, although human resources were adequate, they were unevenly distributed across settings.

References 5-6: 0.90% coverage

Despite rolling out vaccines against COVID-19, testing remains an important measure to control the virus. To effectively be prepared for extensive COVID-19 testing and respond to future outbreaks, the following are recommended: there should be improved political commitments, coordination, and communication with diverse actors to ensure even distribution of all resources across the country; empowerment of community members should be encouraged to develop community-oriented pandemic Frontiers in Public Health | www.frontiersin.org 1 July 2022 | Volume 10 | Article 908410 Institute of Health Partners, Thimphu, Ha et al. Qualitative Study: Factors Inﬂuencing COVID-19 Testing in Ghana preparedness and management of COVID-19 infodemic; investment in strengthening capacity of Good Manufacturing Practice (GMP); incorporation of health policy and systems research (HPSR) into the post-COVID-19 pandemic recovery process and future pandemic preparedness.

Reference 7: 1.28% coverage

Community Leadership The majority of the participants agreed that strong community leadership played an important role in distributing personal July 2022 | Volume 10 | Article 908410 Ha et al. Qualitative Study: Factors Inﬂuencing COVID-19 Testing in Ghana protective equipment (PPE), sensitizing health protocols and encouraging vulnerable groups to actively engage in the community to ﬁght against COVID-19. According to our ﬁndings, community leaders and members played a critical role in successfully managing the pandemic through close collaboration with the Ghana government, health institutions, non-governmental organizations (NGOs), and other faithbased organizations. Furthermore, community leaders, including religious leaders, helped provide public health education for members of their community using their local languages. This enabled community members, especially the illiterate, to understand the COVID-19 burden and the consequences of not adhering to health protocols. “Many religious leaders from the churches are currently educating people to follow all COVID-19 protocols, such as wearing masks, washing hands, and getting tested on the virus. We are doing our best to educate our members to follow the protocols and get tested timely. We also help them to disseminate identiﬁed information related to availing testing”- Community Leader 1.

Reference 8: 1.00% coverage

Multisectoral Partnerships and Collaboration Our ﬁndings revealed a high level of multisectoral partnership and engagements of diverse stakeholders, such as intergovernmental organizations, religious institutions, civil society, and the private sectors. The study participants recounted how engagement with the private sectors for ﬁnancial and material support enhanced COVID-19 testing capabilities, including setting up major testing centers across the country and ensuring testing kits and supplies. For example, Nyaho Medical Center and Frontiers Health Care Services are major private testing centers in the country. The development partners also assisted with the provision of laboratory equipment and the logistics for testing. “. . . we have been supported by various private companies, NGOs, and ministries. The private companies and some NGOs helped some vulnerable groups with health supplies and funds for COVID19 testing. My institution was also given some ﬁnancial and material support from them, such as reagents forCOVID testing.”— Laboratory Manager 1.

References 9-10: 0.86% coverage

Human Resources The study participants indicated that there are suﬃcient health professionals, training programs, and platforms to enhance the capacity of the health workforce to meet up to tasks fully. Also, the interviews highlighted that there were several incentive packages for health workers, including insurance packages and tax relief (19). According to the opinions of some participants, this enabled them to exhibit a positive attitude at work. Also, the training improved the conﬁdence level and interpersonal relationships between health professionals and their clients. “We have been provided extensive training and go through a series of training for 3 weeks or 1 month. We learn everything from whole processes of sample collecting to how to make patients calm Frontiers in Public Health | www.frontiersin.org 5 down to make sure that they are comfortable when taking a nasal pharyngeal swab.” - Laboratory Scientist

Reference 11: 0.81% coverage

Financial and Material Resources Provision of ﬁnancial resources is the key to ensuring adequate health supplies to foster a timely response to COVID-19. The participants mentioned the commitments of the Ministry of Health (MoH) to ensuring adequate ﬁnancial and material resources in upscaling testing. They further emphasized that support from individual donors, international organizations, and NGOs enabled the government to achieve this goal. “We did not have enough testing centers and PPE at the beginning of the pandemic. But, now, we have enough facilities, adequate PPE, and other consumables supported by the Ghana government, international organizations, and other donors for COVID-19 testing. For example, the Ghana airport testing center has been recently established and oﬀers COVID testing at the Kotoka International Airport.”—Laboratory Manager 2

Reference 12: 0.75% coverage

Furthermore, most of the participants emphasized that the availability of more testing centers, infectious disease centers, sustainable funding initiatives, such as the establishment of the COVID-19 National Trust Fund (CNTF), and funding programs for enterprises all contributed to successful COVID-19 responses in Ghana, including testing. A senior oﬃcial at MoH mentioned that “We established COVID-19 Alleviation and Revitalization of Enterprises Support (CARES) program to mitigate the impact of the pandemic on the livelihoods of Ghanaians and support businesses and workers, and introduced a package of economic stimulus measures called the Coronavirus Alleviation Programme (CAP) to formulate and implement the COVID-19 preparedness and response plan, tracing, testing, and treatment.”

References 13-14: 0.46% coverage

Surveillance Systems The participants revealed the establishment of a central procurement system, and a surveillance system was vital to COVID-19 preparedness and response, and provision ofessential services. A senior oﬃcer at MoH said, “We have a good surveillance system to rapidly detect, test, and manage cases to monitor the virus, and Ghana Health Service can see all available data at one glance using it. We also have a good, systemized procurement system through procurement agencies.”

Reference 15: 0.55% coverage

Furthermore, real-time surveillance of reported COVID-19 infections has been the key to the global pandemic response. Many tools, devices, and apps have supported surveillance in Ghana (20). The participants emphasized the role of the centralized data reporting system in Ghana called Surveillance Outbreak and Response Management and Analysis System (SORMAS). According to interviewees, the systemenabled health professionals to identify defaulters and get them tested. They also pointed out that it helped monitor stock to avoid shortages of materials for testing, such as test kits and PPE.

Reference 16: 0.32% coverage

“The Ghana Health Service traces all positive cases based on collected data. It helped connect all labs across districts to access to July 2022 | Volume 10 | Article 908410 Ha et al. Qualitative Study: Factors Inﬂuencing COVID-19 Testing in Ghana it, also, helped detect, investigate, and control the virus in the long run.”—Laboratory Manager 3

Reference 17: 1.07% coverage

Inadequacy of Human Resources (Testers) to Respond to Growing Needs Interestingly, 55% of the participants agreed that the government’s eﬀective interventions in the health sector helped bridge the gap between demand and supply of human resources, while the same proportion of the participants (55%) complained that, despite the government’s eﬀort, there is still an issue of limited human resources, especially in COVID-19 testing centers. A laboratory manager recounted, “We have only one person at the lab who runs the test. Despite our support, he ran samples until late. I also feel too exhausted and tired when testing many people. The human personnel is fewer. One of the reasons is that some people think it is too dangerous, and, maybe, they can use the same expertise to bring more changes in the science ﬁeld, Ghana.” Some health professionals also revealed that they experience high stress and burnout due to a heavy workload during the prolonged pandemic. A nurse said, “I think we have been demotivated for the past years. We are risking our lives during the pandemic, and, sometimes, it makes us miserable and stressful despite our passion for patients.”

Reference 18: 1.90% coverage

Financial and Material Resources Barrier to Testing According to ﬁndings of the study, poor infrastructures, such as low internet connectivity and an inadequate transportation system, posed a challenge to eﬀective delivery of collected blood samples, thereby delaying test results and data loss. Again, there was diﬃculty in transporting tools and equipment due to the poor roads. “The main issue is logistics and transport to deliver blood samples to national labs in Accra (the capital city and where testing labs are located). When we were supposed to leave to take samples outside from the community, the car wasn’t ready. This was the Frontiers in Public Health | www.frontiersin.org 6 challenge during the collection of samples and taking samples to Accra.”—Laboratory Manager 4 The Ghana government received COVID-19 medical supplies from international organizations to be given to hospitals and clinics to scale up the COVID-19 response eﬀort in 2021 (21, 22). However, the majority of the participants asserted that the cost of being tested was high for most people, thereby deterring people from being tested. A laboratory scientist mentioned, “The government should do something about COVID-19 tests by reducing the price or making it free for the ordinary people because most people who do not have a prescription from doctors are reluctant to pay expensive testing fees and get tested.” Also, the participants indicated that the high dependency on imported reagents and consumables from other countries poses a challenge to an eﬀective response to the growing threats, especially whenever such products become scarce in the donors countries. According to the participants, this contributed to the occasional shortages of material resources, such as reagents and testing kits. A researcher at the medical research institute reported, “There is, currently, a shortage of health supplies for testing. This occasionally happens when more people want to get tested and know their status. So, securing enough testing kits and reagents is very important.

Reference 19: 0.79% coverage

Service Delivery Ghana’s health care structure is regarded as well developed compared to other countries in sub-Saharan Africa (4). There are ﬁve levels of providers, including health posts, health centers and clinics, district hospitals, regional hospitals, and tertiary hospitals, to improve accountability to local population, eﬃciency in service provision, equity in access and resource distribution, and increased resource mobilization (26). However, this decentralized system and its functions have not been as eﬀective as they could have been in the outskirts of Accra. A nurse working at a remote hospital fromGreater Accra said, “Like mining town, everybody says they do not have equal opportunities to access good healthcare services compared to the capital city, Accra. Full attention and support for the people in rural areas is really needed.”

References 20-21: 1.31% coverage

As vaccines are rolled out, testing will continue to play a vital role in controlling COVID-19. The main reason is that testing, followed by contact tracing and isolation of those with positive test results, will promptly allow health professionals to monitor the dynamics of the pandemic. Moreover, according to our ﬁndings, COVID-19 testing is still of particular importance to eﬀectively controlling the transmission of the virus in Ghana. Most of the participants conﬁrmed that testing, as an important prevention measure, should be secured with adequate resources and stable health systems. Also, good health governance and leadership, eﬀective resource management, and digitalized information system are successful factors inﬂuencing extensive COVID-19 testing. However, upscaling testing capabilities and facilities is faced with several bottlenecks, such as uneven resource distribution, COVID-19 infodemic, and constraints to service delivery. From the analysis, multilateral cooperation and joint partnerships with diverse stakeholders will play a critical role in facilitating active community participation, investment in GMP, and multilateral political commitment in taking bold actions to build strategies to respond to emerging pandemics. Also, a new research area, HPSR, will be a stimulus for many countries to restructure and develop stronger health systems for future pandemics.

[Files\\Included articles\\Decision-takers_Attitudes_Towards_SARS-CoV-2_Self](bfbb18b7-0c58-4768-9f45-b90bd8eb782a)

1 reference coded, 0.05% coverage

Reference 1: 0.05% coverage

nd because of the healthcare professionals’ attitudes.

[Files\\Included articles\\Diagnostic radiographers’ experience of COVID-19, Gauteng South Africa](3ee8a49c-e68a-4298-a8e2-435fee6c4e53)

4 references coded, 3.94% coverage

Reference 1: 1.58% coverage

New work ﬂow and operations Medical imaging departments' operations were affected by the decrease in imaging referrals at the onset of the lockdown.49 Even though it has been viewed as an opportunity to address “overuse and overdiagnosis”,50 the reduction in imaging referrals, has resulted in some radiographers in the private sector experiencing pay cuts: “…since the lockdown patients were advised not to come to the hospital unnecessarily and with that in private sector, we were affected economically. Less patient ¼ less proﬁt to the company then salaries we cut off” R34 Radiographers' experiences conﬁrm the introduction of new work ﬂow and operations as well as the need to conform to new changes.26e29 The quotes below convey radiographers’ experiences of the changes: “A lot of protocols and precautionary steps taken. Even with all that, people are still confused about what to do, even staff members of other disciplines.” R17 “In my workplace we do not have a set protocol in place for suspected and conﬁrmed cases that are understood by everyone, it sometimes feels like protocols change every day based on who is in charge on that particular day.” R3 “Exhausted from shift changes. Getting irritated with rules that don't make sense.”R59

Reference 2: 1.00% coverage

The South African Department of Health and HPCSA provide guidelines for health practitioners and hospitals with the caveat of them being evolving documents.51e53 Transitioning in ﬂuid contextual circumstances requires constant evaluation of the effectiveness and efﬁciency of work ﬂow and operations. However, success of implementation is embedded in the staff's understanding of the changes effected and the need for the changes20 Similar to radiographer 59 (R59), more radiographers commented on changes to their allocations: “During this pandemic we've also had to work extended hours at the hospital (12 hour shifts for 7 days and then 7 days off which I feel also adds to the exhaustion.” R10 “… So my experience has been okay and at times difﬁcult as we divided ourselves into teams” R7

Reference 3: 0.57% coverage

Radiographers shared their experience of medical imaging de- partments implementing stringent infection control measures that affected work ﬂow and operations.26e28,56 Their responses ranged from having access to sufﬁcient PPE to lack of PPE; lack of disinfectants as well as delayed testing and provision of results.60 Radiographers’ experiences of infection control measures are shared through these direct quotes: “… At least we have PPE.”

Reference 4: 0.78% coverage

We have had to ﬁght tooth and nail to receive PPE. There is a shortage of surface disinfectants. There is no regular screening at the gate for the staff. The backlog ofgetting Covid 19 results is also tiresome.” R4 “Horrible … Lack of PPE!”R16 and R39 “We wear our own fabric masks and do not have much else in the way ofequipment. When some ofmy colleagues had contact with positive cases, they didn't test or quarantine any of us.”R48 “There is a lot of negligence in terms of proper isolation and treatment ofCovid-19 patients. The hospital I'mworking at is not Covid ready and many of the staffmembers here do not feel safe.” R5

[Files\\Included articles\\Implementation of a modified drive-through sampling strategy for SARS-CoV-2-the Nigerian experience](5a4904fa-bf54-46ed-8954-5f5dfa243c8b)

5 references coded, 4.40% coverage

Reference 1: 0.54% coverage

Turn-around time for sample collection and test results: the time stated by clients for sample collection was between fifteen minutes to thirty-five minutes while the turn-around time to obtain results was between twenty-four hours to 72 hours. A respondent who received the result some days after felt that the delay was probably attributed to a backlog.

Reference 2: 0.91% coverage

Also, they believe that security officials should be educated and trained to know how to respond during outbreaks. Respondent 10 stated, ‘I was stopped by an officer on my way to get tested, I explained that I was going for a test and the officer continued to probe to ask what test I was going for, when I told him it was to test for Corona, he asked me how come?’. I felt he was intruding in my private life. Another officer asked me where I was going to, when I brought out my phone to show him the mail sent inviting me to do the test, he thought I was trying to record him and said I should go, that he did not want any trouble.

References 3-4: 1.07% coverage

Overall experience with the drive-through centre: all the clients were impressed and gave a rating of between eighty to ninety-nine point nine per cent. Members of staff were super professional, adhered to safety precaution methods, and were helpful. It was a good initiative from NIMR to test. Respondent 6 stated ‘The NIMR staff are very professional, right from the gate our car was disinfected, we were given face masks, information about the test was provided and the test was done on time’. Another respondent stated, ‘I was scared to take the test but when I came to NIMR, they spoke to me and made me feel comfortable and relaxed, also I spoke to a counsellor who reassured me of the process’ (Respondent 4).

Reference 5: 1.87% coverage

Walkthrough Experience: some of the clients who had to come to the centre by public transport stated that the hitches experienced in coming to the centre were: i) Trekking a distance and long waiting time to get transportation to come to the center. ii) Getting a means of transport to access the centres. iii) Engaging in periodic checks by law enforcement officers who in some cases will ask for some form of evidence. A few had to explain to the officials that they had been exposed and had reached out to relevant institutions in which they were invited/going to take a test. With regards to the process, the respondents stated that when they got to the center, they were decontaminated/an official spoke to them and then briefed them on the processes to expect. Describing the process, the clients stated that from the entry point, they were directed to the wash station to wash their hands then they were moved to Identity verification station and the point where they filled a form from the Nigerian Centre for Disease Control, then a lab bottle was filled and the specimen collected. Also, on their way out they were sprayed again (decontaminated) before leaving the testing center and then had to wait for their results to come in via the mail.

[Files\\Included articles\\Innovations, contestations and fragilities of the health system response to COVID-19 in the Gauteng Province of South Africa](a637b471-34ef-4fe8-899e-0f210e67bcc1)

4 references coded, 2.03% coverage

Reference 1: 0.43% coverage

In Gauteng, there was recognition ofthe need to ensure financial, human and other resources to meet the increased demand for hospitalisation and quarantine. The Gauteng Provincial Government allocated an additional R4 billion (~267 million US$; $1 = R15) to the health sector for the appointment ofadditional staff, the procurement ofPPE, and the upgrading or building ofadditional infrastructure. Many key informants viewed this significant injection offinances in a positive light. The response to COVID-19 was comprehensive. We were able to get additional finances. We also took the decision to revamp the current infrastructure, using alternative building technology (KI 4, Executive Manager). The support was palpable on the ground. . .we were given R14 million (~$933 000) to beefup current staffing levels. The budget mitigated staffshortages especially with the opening ofnew wards (KI 8, Central Hospital Manager).

Reference 2: 0.74% coverage

The document analysis revealed the establishment ofa separate Public Health Stream within the health department that presented detailed reports to the Provincial Advisory Council, which in turn reported to the overall governance structure, namely the Provincial Coronavirus Command Council [45]. On paper, there were detailed strategies and procedures that dealt with epidemiology and surveillance, community mobilisation, education and advocacy, and case management and public health training [45]. However, key informant interviews demonstrated a lack ofclarity or knowledge on the provincial COVID-19 testing strategy. There was failure to share the Provincial Testing Strategy, especially on the employee testing. There was also non-compliance with testing guidelines e.g. random testing ofcommunities without following the guidelines, leading to conflict with our laboratory team (KI 20, Technical Support/ Research/ Academic). Frontline staff highlighted the challenges experienced with laboratory testing. While we were managing the pandemic, we had a lot ofareas that were not in sync, so this is an area we can improve e.g. the laboratory tests, I don’t know where the problem was, but we PLOSONE | https://doi.org/10.1371/journal.pone.0261339 December 17, 2021 17 / 28 PLOS ONE Health system response to COVID-19 in the Gauteng Province of South Africa had a lot ofinstances where people would be reported as negative or positive, but there would be missing information—no addresses and contact numbers, so it made contact tracing difficult (KI 34, District Health Manager)

References 3-4: 0.85% coverage

Theme 4: Under-investment in the health workforce. Although many key informants expressed appreciation for the additional funding and staffappointments as part ofthe COVID-19 response, a recurring theme in the interviews was the perceived under-investment and insufficient focus on the health workforce, especially on frontline staff. Some key informants were ofthe opinion that there was lack ofprioritisation ofhuman resources and that the health department did not demonstrate appreciation of staff, despite health workers risking their lives to do their duties. Doctors are burnt out, nothing you do really is recognised, morale is low, and there is poor support, lack ofequipment, unfilled trolleys, staffshortages, having to run around for stock . . .it a very draining emotional experience (KI 36, Central Hospital Clinician). Health care workers contracted COVID 19 and some lost their lives. We should have given a little more attention to frontline health workers e.g. buses provided for travel so that they don’t have to contract COVID, more emotional (psychological) support (KI 32, PHC Facility Manager). They lamented insufficient involvement offrontline staff in the pandemic response, or in certain decisions, such as putting up separate tents for COVID-19 positive patients. We should have been involved in discussions around how oxygen will be supplied to the tent, and that is a problem. . ..sometimes you run out ofoxygen during a resuscitation (KI 36, Central Hospital Clinician). Poor or sub-optimal human resource management exacerbated the perceived under-investment in the health workforce. COVID-19 exposed poor managers who often took out their frustration on ordinary health workers. This was in contrast to those managers who were able to respond to pressure (KI 22, Professional Association).

[Files\\Included articles\\Perceptions and opinions of Nigerians to the management and response to COVID-19 in Nigeria](05196e70-6ed3-4244-806c-4e0bd5d8c317)

8 references coded, 3.73% coverage

Reference 1: 0.17% coverage

molecular testing should be diversified with all Federal Medical Centres (FMCs) having a laboratory dedicated to COVID-19.

Reference 2: 0.64% coverage

Respondents suggested that in line with measures taken by other countries, the Federal Nigerian Government should provide life insurance cover with critical illness and total temporary disability for healthcare workers in Nigeria with a sum assured of not less than 25,000,000 Naira per life (65,000 US dollars); provide adequate supplies in terms of social care, stationery and laboratory supplies, increase COVID-19 testing centres and community based learning programmes.

References 3-4: 0.44% coverage

Some respondents thought that healthcare personnel handling HIV antiretroviral therapy (HIV ART) should be trained in COVID-19 testing, with Obinna Ositadimma Oleribe et al. PAMJ - 40(185). 26 Nov 2021. - Page numbers not for citation purposes. 5 Article linkage to care and information management for reference laboratories.

Reference 5: 1.23% coverage

its infrastructure”: many respondents believed that the COVID-19 outbreak revealed the failure of the healthcare system and its lack of capacity to handle emergencies, and suggested “The Nigerian healthcare system should be properly funded to ensure effective service delivery.” To them, there were not enough healthcare workers to handle the problem, and those available were not properly trained in pandemic management. Moreover, it was felt that there was not enough PPE, infrastructure and consumables to handle the pandemic. The respondents therefore asked for people investment in healthcare to rebuild the healthcare system for present and future disease outbreaks. In addition, to achieve the set objectives, respondents suggested that the Government should engage professional associations like the Nigerian Medical Association (NMA) in the planning and implementation of the various control measures.

References 6-7: 0.65% coverage

“Depoliticising the epidemic”: some respondents believed that the epidemic was politicized. To this end, they asked for depoliticization of the COVID19 control process. One said, “We may not have the luxury of large isolation centres to cater for all the people who will test positive, but we can make the treatment protocols available in every locality, so that the majority of people can be treated in their homes. This should be possible if testing kits are available for early detection.”

Reference 8: 0.59% coverage

ow COVID-19 testing rates, inadequate facilities, a lack of enforcement of movement restriction, profiting from COVID-19 by media and other stakeholders, the nonenforcement of interstate travelling by security agencies, lack of trust for local drugs, violation of lockdown rules by Nigerians, hunger increase, and ineffective distribution of relief materials were some of the things that were perceived to be lacking by the respondents.

[Files\\Included articles\\Preparedness and response to covid-19 in Woreta Town, North West Ethiopia](bf44178e-777e-489a-ba3d-c59766ae3cd6)

12 references coded, 3.58% coverage

References 1-2: 0.69% coverage

Public health interventions This paper addressed the preparedness and response measures undertaken in the health setting and in the community. It also focused on activities preformed to increase the community’s COVID-19 practice. The study analyzed the activity of all actors including the local government, the task force, community members and health workers. The collected data showed that the study area had no even a primary hospital, let alone, a general hospital. Three years were passed without ﬁnishing the building of a primary hospital under construction. There were no functional quarantine and isolation centers. The less developed health system in Woreta demanded to have a dual referral system in which specimens were referred to Bahirdar city for laboratory diagnosis while infected people were referred to Debre Tabor for isolation and treatment. Besides, the non-fulﬁllment of the following basic pillars shows the low preparedness and response in the study area.

Reference 3: 0.17% coverage

Community engagement Denial, Suspicion “Extension workers come and measure body temperature and ask if there is a recent cough or fever. Religious leaders also teaching in churches to keep distance, but no one practices it”, a resident in 04

References 4-5: 0.43% coverage

Rapid response COVID-19 Rapid Response Team (RRT) was in place. The team comprised nine health experts. It was led by a trained Public Health Emergency (PHEM) Oﬃcer. It collects and investigates rumors; assesses corona virus sign and symptoms; deﬁnes cases; identiﬁes suspects; traces contact and lists them; advises self-quarantine or refers to an isolation center. The activities were affected by lack of PPEs, equipment and services. Only one ambulance was giving transport service for all health-related tasks. It transported RRT members, referred non-Covid-19 patients, specimens and medical supplies.

Reference 6: 0.61% coverage

urveillance Covid-19 surveillance had been conducted in Woreta. During the home-to-home visit, the health workers were measuring resident’s body temperature and asking them if they had any Acute Respiratory Infection (ARI) or experienced its symptoms such fever and cough. “Extension workers come and measure body temperature and ask if there is a recent cough or fever”. A resident in kebele 04 The RRT leader stated that, prior assessment and screening was conducted in two rounds from May to June 2020. In the third round, Community Based Surveillance (CBS) was done starting from 11 August 2020. The CBS was part of a national 6 A .A . Mohammed Scientiﬁc African 14 (2021) e01037 program simultaneously implemented throughout the country for one month. It was intended to boost COVID-19 test and public engagement in reporting events having public health signiﬁcance.

References 7-8: 0.29% coverage

Three concerns were raised in relation to surveillance. First, it was not supported by vigorous rumor collection. Sec- ond, surveillance data was not admitted to epidemiological analysis and projection. Third, persons identiﬁed for probable cases were allowed to give samples after a delayed period of time due to lack of kits. To the contrary, some of them hide themselves when they were wanted for giving specimen.

References 9-10: 0.70% coverage

Samples were selectively taken. Regular sites of sample collection included; the bus station, the dry port, the food re- serve station and special forces’ camp. Recently, sample collection was expanded to hotels, and banks. Sometimes, suspects identiﬁed during home visit campaign hide themselves. The specimen collection was interrupted many times due to lack of kits. When kits were available, up to 80 samples were daily collected within the town. According to the RRT representative, the total number of tests within the CBS month (from mid-August up to early September) was 1200 and total caseload was nine until 18 September 2020. Surveillance and laboratory activities were accompanied by continuous contact tracing tasks. “Starting from day one (June 16/2020), when the ﬁrst infected person was identiﬁed in the bus station, contact tracing has been made. A total of 46 close contacts were identiﬁed from the ﬁrst case and two close relatives of that individual were tested positive.” A PHEM Oﬃcer.

Reference 11: 0.32% coverage

Laboratory COVID-19 diagnostic services were not started in the study area because the laboratory in Woreta had no necessary kits. In collaboration with higher level structures, specimens had been collected and referred to Bahirdar city for molecular laboratory test. According to an interviewee, the results were told to the people within two or three days. Based on the test result, people are referred to isolation centers after their contacts were traced.

Reference 12: 0.36% coverage

Logistics, procurement and supply management Due to lack of budget and weak supply chain, the provision of medical equipment was short and irregular. For instance, there was a low stockpile of hand hygiene materials (alcohol and sanitizer). There was no supply of medical masks and full protective gowns as well as materials to furnish quarantines and isolation centers. There was no consistent supply of laboratory kits from upper government organs. Therefore, the RRT got diﬃculty to collect COVID-19 samples.

[Files\\Included articles\\Self testing for COVID 19 in Durban and Eastern Cape South Africa a qualitative inquiry targeting decision takers](6ca4769f-dd80-4c55-b8ad-484b514bf85b)

6 references coded, 1.85% coverage

Reference 1: 0.58% coverage

In Durban, informants identiﬁed a public–private sector dichotomy. Particularly among RCSs, testing services were described as distributed along a gradient of access, convenience, and cost, with free testing available at government facilities but with long waiting times. In contrast, private testing was considered by RCSs more available but also less convenient due to its cost. Accessing testing was considered burdensome and ‘dangerous’, given fears that SARS-CoV-2 could be transmitted while waiting in queues. In Durban…the only options available are to go to a test center, either to government test centers, which are free and slow, or go to private ones, which are fast and expensive. (RCS, 55, male

Reference 2: 0.21% coverage

In King Sabata Dalindyebo, hospitals, private clinics, and consultations of general practitioners were identiﬁed as the primary testing sites available. One exception was mentioned by a male RCS who recounted how a ‘testing team’ had come to his workplace.

Reference 3: 0.31% coverage

The barriers identiﬁed to conventional facility-based testing were diverse. The expense of COVID-19 testing was mentioned by all groups, either incurred from lost wages while waiting in queues for government testing or the cost of private testing. The cost of transportation was a barrier in King Sabata Dalindyebo, where testing facilities are geographically dispersed.

Reference 4: 0.27% coverage

here was consensus that the health system was under-resourced prior to the pandemic. A long-lived lack of resources, including insuﬃcient personnel and transportation for providing community-based care, was commonly cited as the reason for the system’s inability to reach everyone who requires testing in current times.

Reference 5: 0.14% coverage

RCSs feared that the same barriers to facility-based testing would apply to self-testing. Financial considerations could cause diﬃculty in obtaining self-testing.

Reference 6: 0.34% coverage

Regarding locations to obtain a self-test, informants generally agreed that self-tests should be widely available, including at clinics, pharmacies, and hospitals. Grocery stores were also considered suitable, although in non-healthcare settings some informants felt that health authority representatives should be present to advise individuals on follow-up actions to take if their self-test was positive.

[Files\\Included articles\\Stakeholders perspective of, and experience with contact tracing for COVID-19 in Ghana- A qualitative study among contact tracers, supervisors, and contacts](d64e5f0f-8bc1-4eaa-89c9-12808f4ee8d2)

16 references coded, 9.69% coverage

Reference 1: 0.03% coverage

elays in receiving test results,

Reference 2: 0.09% coverage

Improving coordination and quick release of test results to contacts is necessary for COVID-19 containment.

Reference 3: 0.80% coverage

Identification of contacts for COVID-19 Contact tracers and supervisors defined a contact as anybody who shares any form ofclose physical space with a confirmed COVID-19 case/person. The person could be a friend, colleague worker, bordered the same transport, sleeping in the same house, house-keeper ofa confirmed case, or provided health care to the cases. The proximity ofthe confirmed case to the contact is used to determine the risk profile ofthe person. A supervisor shared his views on who a contact is as follows: “A contact is someone who has physically interacted with a person who has tested positive. We have two contacts–close-contact and distant contacts. We also use the WHO approach. When you identify someone as a suspected case and is confirmed to have COVID-19, a health worker goes to the person to do the listing. We help the person think about all his/her activities two days before the test was confirmed. This is done day by day” (S003)

Reference 4: 0.79% coverage

Contacts for COVID-19 are identified through case investigation. This involved taking the history of all confirmed cases, his/her movements, and activities. It gives a clear indication of the people the case would have encountered as explained by one supervisor: “We use case investigation forms-travel history, attending a health facility and treated for any illness prior to being confirmed” (S002) Wherever a case is confirmed, all people who live in a two kilometres radius ofthe residence ofthe confirmed case are screened. This is done as a form ofenhanced contact tracing. One supervisor’s explanation is as follows: “We introduced a strategy in our enhanced contact tracing and testing. Because some confirmed cases are asymptomatic, we take a certain radius ofall people within the vicinity ofthe confirmed case. This is done with the beliefthat the confirmed case may have closely related to people within this radius” (S001)

Reference 5: 0.54% coverage

A line list of all the contacts is done, and initial samples are taken for the test. After that, daily monitoring is done. This monitoring is done twice daily—morning and evening. During the contact monitoring, the temperature ofthe person, other socio-demographic and illness history are taken. After fourteen days, the samples are taken for a repeat test to be conducted. A supervisor shared his experience on how monitoring is done: “The monitoring is supposed to be done twice a day, but in many instances, the monitoring is done once a day. During the contact, the contact tracer fill the case-based form and checking oftemperature” (S005)

Reference 6: 2.08% coverage

Selection, training, and deployment of contact tracers The study showed that contact tracers included public health physicians, field epidemiologists, disease control officers, nurses, and volunteers. Contact tracers were selected based on their professional background and those involved in already existing surveillance chains for other endemic infectious diseases. At the community level, there are community health nurses as contact tracers. At the district level, there are disease control officers with epidemiologists and clinicians at the regional level. These cadres ofhealth workers already have prior training on filling case-based forms. All the contact tracers were health workers with some level oftraining on infectious diseases as confirmed by some supervisors in the following statements: “We look for a cadre ofstaffwho qualify to be contact tracer. We look for people with basic training in public health, understand the health system. Each district identify their contact tracers” (S004) “All our contact tracers are health workers who have formal training” (S003) Contact tracers in this study corroborated that they had received training before they were deployed. A contact tracer shared his view about the training as follows: “We were given some basic training on the contact tracing. We were taken through the casebased forms and the dos and don’ts in contact tracing. We were informed about how to protect ourselves from infection, not eat or drink anything from a contact” (CT003) The content ofthe training includes signs and symptoms ofCOVID-19 and completing the symptoms diary and daily monitoring ofcharts: “We go through the symptom’s dairy and daily monitoring charts with them. These charts come in two forms, paper-based and electronic” (S002) The training also covered the definition ofcontact, how to do contact listing and tracing. The tracers were trained on the use ofthe infra-red thermometers, data collection and use of personal protective equipment (PPE). The contact tracers were also trained on how to use the Surveillance Outbreak Response Management and Analysis System (SORMAS) and ARC GIS software. A supervisor and contact tracer shared their experience on the content ofthe training: “We trained all the contact tracers. They were trained before first confirmed cases in the country. . .after the detection ofthe first two cases, another training was done before they were deplored” (S001).

Reference 7: 1.04% coverage

“We received training twice, the first one occurred before the first cases and later after the first two cases were confirmed. We were trained on the use ofthe SORMAS software” (CT001) Some contact tracers in an interview indicated they had had prior experience in conducting contact tracing. Some were already engaged in contact tracing for diseases such as tuberculosis, Ebola, and other infectious diseases. One contact tracer shared his experience: “I have been involved in contact tracing. During the Ebola outbreak in Liberia, I was involved in contact tracing. So, I have some experience in contact tracing” (CT0015) All contact tracers were unanimous that the training was very useful in empowering them to conduct contact tracing without fear ofbeing infected. “The training actually was very useful. I have learned how to use the SORMAS and GIS software and this would help me in future” (CT0012) Deployment ofcontact tracers was in pairs in order for them to support each other while in the field in terms ofworkload and to remind each other about safety measures while on duty. This was emphasized by a contact tracer: “We move in pairs. That is important as you can be reminded by a colleague about some safety measures” (CT008)

Reference 8: 0.66% coverage

OVID-19 contacts revealed they were delighted with the work ofcontact tracers. To the study participants, the contact tracers were very professional and provided them with the necessary health education and support. All contact tracers also wore a face mask. A contact shared his experience as follows: “They [contact tracers] call to explain to me what they will be doing. . . they came wearing masks and maintained some distance from me. . .in all, I will say they were very professional” (C002) In the same way, contact tracers generally were ofthe view that contacts were very cooperative in their engagements with them: “My experience with all the contacts I have traced so far have been cooperative. They have been able to provide me with all the information I required” (CT0011).

Reference 9: 0.16% coverage

“It was a man and woman; they try to engage you in conversation. They go through the various symptoms, and the temperature check. . .they were calm and provided some psychological support” (C004).

Reference 10: 0.94% coverage

Challenges and lessons learned in the contact tracing Five main challenges were identified; provision ofPPEs, remuneration, and refusal ofsome contact to testing, poor coordination, people not adhering to personal protection etiquettes, and quarantine. Some contact tracers expressed concern about the type and inadequate provision ofPPEs for personal use. During the early stages ofthe epidemic, participants indicated that contact tracers were basically concern with daily monitoring ofcontacts. However, as the number ofcases increased, contact tracers had to take up the responsibility oftaking COVID19 samples. This according to contact tracers, required more advanced PPEs that were unavailable. Two contact tracers shared their views on the provision ofPPE as follows: “We are only given facemask and hand sanitisers, but now some ofus take samples and hence the risk is high. So, we need more advanced PPEs like N95, eye shields and biohazard suits” (CT0011). “We do not have enough PPEs, and you know the work we do is risky. You can get infected because ofdaily interaction with contacts” (CT007)

Reference 11: 0.55% coverage

Participants also revealed details oftheir remuneration. At the beginning ofthe engagement as contact tracers, they were paid daily. However, as the number ofcases increased, more contact tracers were engaged, resulting in delays in payments. Some participants also indicated there was a reduction in the amount they were promised as remuneration even though the workload had increased. The following illustrative quotes support these points. “Initially we were receiving a daily allowance, however, as the number ofcases increased, more people were recruited and it in a way affected our allowance. Now we do not get the daily allowance” (CT012).

Reference 12: 0.12% coverage

“I have been working as a supervisor but, yet to be given the allowance. . . This is negatively affecting the zeal ofcontact tracers” (S003)

Reference 13: 0.70% coverage

Furthermore, delays in getting test results also emerged as one ofthe challenges. This, according to respondents, has resulted in some contacts denying the outcome of tests. This has also resulted in a situation where some contacts refuse to undergo the test. “The delays in getting the test results are affecting the contact tracing. You take the sample, and it takes several days before you inform the person ofthe outcome ofthe test. Because of that, some people will tell you the test results is not for them especially when it is positive” (CT005) “One ofmy staffshared his experience where the contact insulted them and refused to allow them to take samples and complete the case-based forms. We also have contacts who we are unable to trace once the results are back. Others will not also tell you the possible contact” (S006)

Reference 14: 0.31% coverage

astly, participants indicated a lack ofcoordination in the entire COVID-19 response and contact tracing. The contact tracing platform and the testing results were managed by two different groups and platforms making coordination difficult. This was cited as one ofthe reasons for the delays in receiving test results and its associated negative effects on the process.

Reference 15: 0.42% coverage

Participants also raised issues about delays in getting test results and lack ofcoordination. The present study has further shown that respondents perceived delays in getting test results as a significant challenge in the community. According to the study participants, this resulted in some contacts refusing to take the COVID-test or when tested the outcome of test results. It is important for the country to improve on the interval between the taking ofsamples and giving feedback on the results.

Reference 16: 0.44% coverage

Despite the contact tracers and supervisors’ seeming enthusiasm for the work, they were worried about the delay in payment ofremuneration and lack ofcoordination—an essential requirement for the success ofthe entire COVID-19 response. Initially, contact tracers were paid daily but as the number ofcases increased, more people were recruited from within and outside the health system to support the exercise. This led to the delay in the preparation and payment oftheir remuneration which affected their morale to work.
