# Supplementary material for: Factors influencing uptake of COVID-19 diagnostics in Sub-Saharan Africa: a rapid scoping review
Source: PLoS One. 2025 Mar 20;20(3):e0305512. doi: 10.1371/journal.pone.0305512 (PMC11925277; doi:10.1371/journal.pone.0305512)
Supplement: Attachment — Submitted filename: PONE-D-24-22108 2024.docx [file pone.0305512.s007.docx]

Thank you for having me review this important paper titled ‘on Factor influencing uptake of COVID-19 diagnostics 2 in Sub-Saharan Africa: a rapid scoping review 3 Short title: COVID-19 diagnostics uptake in sub-Saharan Africa.

The paper reads well, great analysis and discussion. However, the methodology section seems hurriedly put together yet this is very critical to scoping review. It confuses the reader.

I suggest you organize your presentation in scoping review steps, it will improve clarity.

- Missing, for instance, is the research question (s).
- Besides inclusion and exclusion criteria, need to explain how studies were selected, who or how many researchers conducted study selection, how conflicts were solved, if any.
- Some information in the first paragraph of results should be included in the study inclusion/exclusion, and selection.
- Lewis et al., 2021: qualitative open-ended questionnaire- is this qualitative or mixed method?
- Why is the scoping review period up to July 2023?
