# Supplementary material for: Factors influencing uptake of COVID-19 diagnostics in Sub-Saharan Africa: a rapid scoping review
Source: PLoS One. 2025 Mar 20;20(3):e0305512. doi: 10.1371/journal.pone.0305512 (PMC11925277; doi:10.1371/journal.pone.0305512)
Supplement: Attachment — Submitted filename: Response to Reviewers.docx [file pone.0305512.s009.docx]

Ermel Johnson, MD, MPH, PhDc
Academic Editor
PLOS ONE

22 January 2025

**Subject: Rebuttal Letter for Manuscript PONE-D-24-22108**

Dear Dr Ermel,

We sincerely appreciate the constructive feedback provided by the reviewers and thank you for the opportunity to revise our manuscript. Below, we provide detailed responses to each comment, addressing both major and minor revisions as requested. We believe these revisions have significantly strengthened our work.

**Major Revisions**

**Methods**

**Comment 1**: In the methods section, please provide information on how duplicates were removed during the search, how many researchers extracted the data independently, and how the discrepancies were handled.
**Response**: Thank you for raising this concern. We have revised the “Study Selection and Data Extraction” section to include these details. Specifically, duplicates were manually removed by one author, and data extraction was collaboratively conducted by three authors, enabling real-time consensus and eliminating discrepancies.

**Results**

**Comment 2**: Provide the results of the appraisal of the studies.
**Response**: Results of the study appraisal were included in the initial submission as Supplementary Material 2. The appraisal scores, ranging from 1 (low quality) to 4 (high quality), have been clearly outlined in the updated submission for clarity.

**Comment 3**: Table 3 should include a column for numerical references.
**Response**: Thank you for this suggestion. We have now included a column for numerical references in Table 3.

**Comment 4**: Key findings in Table 3 should be divided into facilitators and barriers.
**Response**: We appreciate this suggestion. We have now included two additional columns in Table 3: one for “Testing Facilitators” and another for “Testing Barriers.”

**Comment 5**: Provide a table showing themes and subthemes.
**Response**: A new table summarizing the themes and subthemes reported in the results section has been included as per the recommendation.

**Comment 6**: Authors should not rely on a single source for quotes in subthemes and should include quotes for all subthemes.
**Response**: Thank you for this feedback. We have now ensured that all subthemes are supported by quotes, with sources distributed across multiple studies.

**Comment 7**: Avoid mixing numerical referencing with APA/Harvard.
**Response**: The entire manuscript has been revised to use a single numerical referencing style throughout.

**Discussion**

**Comment 8**: Compare results with COVID-19 testing in other LMICs.
**Response**: We appreciate this suggestion and have strengthened the discussion by adding a passage (lines 513–519) that compares findings with COVID-19 testing practices in other LMICs, such as Vietnam and India. These additions highlight shared challenges, such as laboratory infrastructure deficiencies and the role of political leadership, drawing parallels to the SSA context.

**Minor Revisions**

**Introduction and Methodology**

**Comment 9**: Replace “publics” with “the public.”
**Response**: We have corrected this throughout the manuscript.

**Comment 10**: Replace “othering” with “other.”
**Response**: We appreciate the feedback but retained “othering” as it aligns with the social and psychological concept relevant to the context. The concept describes the process of perceiving or treating a person or group as different from oneself. In this context, it refers to communities or individuals perceiving other people or settings to be more vulnerable to COVID-19 and therefore requiring testing more than themselves, and refraining from testing because they perceived themselves to be at lower risk.

**Comment 11**: In line 78, the authors state, ‘Increased availability of diagnostic interventions for COVID-19 (C-19) was….’ COVID-19 is already an acronym, so use it instead of C-19 throughout the manuscript.
**Response**: The manuscript now consistently uses “COVID-19.”

**Comment 12**: Provide reference for lines 103-105.
**Response**: The requested references have been added.

**Comment 13**: In line 199, correct the year for Yamanis reference.
**Response**: Typographical errors have been addressed, and publication years are now consistently formatted in brackets throughout.

**Comment 14**: In line 199, you state, ‘Ha et al., 2022 and Yamanis et al., 20233 described that in areas….’ The publication years should be in brackets throughout the manuscript.
**Response**: Typographical errors have been addressed, and publication years are now consistently formatted in brackets throughout.

**Comment 15**: In line 233, provide reference for Brumwell et al. reference.
**Response**: The missing reference has now been added.

**Comment 16**: In line 240, the authors state, ‘C-19 was more acceptable 240 when spearheaded by people that community members…’ Add ‘testing’ after C-19.
**Response**: We have addresssed this, and ‘testing’ has now been added.

**Comment 17**: Lines 254-260 is a different theme as it does not contain information on novel COVID-19 modalities.
**Response**: A new theme, “Public Health Communication,” has been added to address this.

**Comment 18**: Use numerical references in lines 354-360 and 379-381.
**Response**: The entire manuscript has been revised to use a single numerical referencing style throughout.

**Comment 19**: In line 451, the authors state, ‘The study was enhanced by the systematic searching of several databases to find all relevant studies that satisfied the predefined inclusion criteria.’ Since the authors did not search all databases, saying ‘all relevant studies’ is misleading.
**Response**: Thank you for highlighting this important point. We agree that the original phrasing could be misleading. We have revised the statement to clarify: “The study was enhanced by the systematic searching of multiple databases to identify studies that met the predefined inclusion criteria.”

**Reviewer #2**

We appreciate the positive feedback regarding the relevance of the topic and the presentation of the analysis and discussion sections.

**Comment 20**: Missing, for instance, is the research question (s).
**Response**: We appreciate the feedback. Our research question, “What are the contextual factors influencing people’s decisions regarding COVID-19 testing Sub Saharan Africa?”, has been included in the final passage of the introduction section (lines ), where we have described the purpose of the study: “As part of this work, we conducted a scoping of the contextual factors influencing people’s decisions regarding COVID-19 testing in various settings throughout SSA. This information would support the implementation of the main project.”

**Comment 21**: Besides inclusion and exclusion criteria, need to explain how studies were selected, who or how many researchers conducted study selection, how conflicts were solved, if any.
**Response**: The methodology section now specifies how studies were selected, how duplicates were removed, and how consensus was reached during data extraction (lines 157-168).

**Comment 22**: Some information in the first paragraph of results should be included in the study inclusion/exclusion, and selection.
**Response**: This information has been relocated to the study inclusion/exclusion and selection subsection.

**Comment 23**: Lewis et al., 2021: qualitative open-ended questionnaire- is this qualitative or mixed method?
**Response**: We have clarified that Lewis et al. employed a qualitative approach using an asynchronous opened-ended online questionnaire, where respondents recorded their answers to pre-arranged questions, and responses from purposively sampled diagnostic radiographers were thematically analysed.

**Comment 24**: Why is the scoping review period up to July 2023?
**Response**: The scoping review period was strategically set to follow the global peak of the pandemic when a substantial body of research on COVID-19 testing was anticipated.

We have carefully addressed all reviewer comments to enhance the manuscript's clarity, rigor, and relevance. We trust that these revisions meet the expectations of the reviewers and the journal.

Thank you once again for the opportunity to revise and resubmit our manuscript. We look forward to your feedback.

Yours sincerely,
Mackwellings Phiri
(On behalf of authors)
