# Supplementary material for: Factors influencing uptake of COVID-19 diagnostics in Sub-Saharan Africa: a rapid scoping review
Source: PLoS One. 2025 Mar 20;20(3):e0305512. doi: 10.1371/journal.pone.0305512 (PMC11925277; doi:10.1371/journal.pone.0305512)
Supplement: Peer review history

# Author comment: Author response to Decision Letter 0

**DOI:** 10.1371/journal.pone.0305512.r001

31 May 2024

# Peer review: Decision Letter 0

**DOI:** 10.1371/journal.pone.0305512.r002

**Authors:** Ermel Johnson

24 Jul 2024

PONE-D-24-22108Factors influencing uptake of COVID-19 diagnostics in Sub-Saharan Africa: a rapid scoping reviewPLOS ONE

Dear Dr. Phiri,

Thank you for submitting your manuscript to PLOS ONE. After careful consideration, we feel that it has merit but does not fully meet PLOS ONE’s publication criteria as it currently stands. Therefore, we invite you to submit a revised version of the manuscript that addresses the points raised during the review process. We value the rigour of the articles published in PLOS ONE. The main recommendation relates to your methodology. Please take the reviewers' recommendations very seriously and, above all, follow the relevant guidelines.

Please submit your revised manuscript by Sep 07 2024 11:59PM. If you will need more time than this to complete your revisions, please reply to this message or contact the journal office at plosone@plos.org . When you're ready to submit your revision, log on to https://www.editorialmanager.com/pone/ and select the 'Submissions Needing Revision' folder to locate your manuscript file.

Please include the following items when submitting your revised manuscript:

- A rebuttal letter that responds to each point raised by the academic editor and reviewer(s). You should upload this letter as a separate file labeled 'Response to Reviewers'.
- A marked-up copy of your manuscript that highlights changes made to the original version. You should upload this as a separate file labeled 'Revised Manuscript with Track Changes'.
- An unmarked version of your revised paper without tracked changes. You should upload this as a separate file labeled 'Manuscript'.

If you would like to make changes to your financial disclosure, please include your updated statement in your cover letter. Guidelines for resubmitting your figure files are available below the reviewer comments at the end of this letter.

If applicable, we recommend that you deposit your laboratory protocols in protocols.io to enhance the reproducibility of your results. Protocols.io assigns your protocol its own identifier (DOI) so that it can be cited independently in the future. For instructions see: https://journals.plos.org/plosone/s/submission-guidelines#loc-laboratory-protocols . Additionally, PLOS ONE offers an option for publishing peer-reviewed Lab Protocol articles, which describe protocols hosted on protocols.io. Read more information on sharing protocols at https://plos.org/protocols?utm_medium=editorial-email&utm_source=authorletters&utm_campaign=protocols .

We look forward to receiving your revised manuscript.

Kind regards,

Ermel Johnson, MD, MPH, PhDc

Academic Editor

PLOS ONE

Journal Requirements:

When submitting your revision, we need you to address these additional requirements.

1. Please ensure that your manuscript meets PLOS ONE's style requirements, including those for file naming. The PLOS ONE style templates can be found at 

https://journals.plos.org/plosone/s/file?id=wjVg/PLOSOne_formatting_sample_main_body.pdf and 

https://journals.plos.org/plosone/s/file?id=ba62/PLOSOne_formatting_sample_title_authors_affiliations.pdf

2. Thank you for stating the following financial disclosure:"Funding for this study was received under the STAR COVID-19 grant by UNITAID through Population Services International (grant ref/code: 2017-16-PSI-STAR)." 

Please state what role the funders took in the study.  If the funders had no role, please state: ""The funders had no role in study design, data collection and analysis, decision to publish, or preparation of the manuscript."" 

If this statement is not correct you must amend it as needed. 

Please include this amended Role of Funder statement in your cover letter; we will change the online submission form on your behalf.

3. Your ethics statement should only appear in the Methods section of your manuscript. If your ethics statement is written in any section besides the Methods, please move it to the Methods section and delete it from any other section. Please ensure that your ethics statement is included in your manuscript, as the ethics statement entered into the online submission form will not be published alongside your manuscript. 

[Note: HTML markup is below. Please do not edit.]

Reviewers' comments:

Reviewer's Responses to Questions

**Comments to the Author**

1. Is the manuscript technically sound, and do the data support the conclusions?

The manuscript must describe a technically sound piece of scientific research with data that supports the conclusions. Experiments must have been conducted rigorously, with appropriate controls, replication, and sample sizes. The conclusions must be drawn appropriately based on the data presented. 

Reviewer #1: Yes

Reviewer #2: Yes

**********

2. Has the statistical analysis been performed appropriately and rigorously? 

Reviewer #1: N/A

Reviewer #2: Yes

**********

3. Have the authors made all data underlying the findings in their manuscript fully available?

The PLOS Data policy requires authors to make all data underlying the findings described in their manuscript fully available without restriction, with rare exception (please refer to the Data Availability Statement in the manuscript PDF file). The data should be provided as part of the manuscript or its supporting information, or deposited to a public repository. For example, in addition to summary statistics, the data points behind means, medians and variance measures should be available. If there are restrictions on publicly sharing data—e.g. participant privacy or use of data from a third party—those must be specified.

Reviewer #1: Yes

Reviewer #2: Yes

**********

4. Is the manuscript presented in an intelligible fashion and written in standard English?

PLOS ONE does not copyedit accepted manuscripts, so the language in submitted articles must be clear, correct, and unambiguous. Any typographical or grammatical errors should be corrected at revision, so please note any specific errors here.

Reviewer #1: Yes

Reviewer #2: Yes

**********

5. Review Comments to the Author

Please use the space provided to explain your answers to the questions above. You may also include additional comments for the author, including concerns about dual publication, research ethics, or publication ethics. (Please upload your review as an attachment if it exceeds 20,000 characters)

Reviewer #1: Thank you for an opportunity to review this manuscript. This manuscript is of relevance to healthcare practitioners and policymakers as its findings can be used to prepare for future pandemics. The introduction orients the reader to the topic. The methodology is clear but needs improvement. The results and discussion sections should also be improved. The conclusion is based on the findings. In-text referencing was not consistent.

MAJOR REVISIONS

Methods

I. In the methods section, please provide information on how duplicates were removed during the search, how many researchers extracted the data independently, and how the discrepancies were handled.

Results

1. Provide the results of the appraisal of the studies.

2. Table 3 should provide a column for the numerical references.

3. In table 3, the key findings should be divided into facilitators and barriers, and these should be the columns in the extreme left.

4. Provide a table showing the themes and the subthemes.

5. Authors should not use one source for quotes in their subthemes.

6. Each subtheme should be supported by a quote, not just a selected few.

7. Authors should not mix numerical referencing and APA/Havard.

Discussion

8. Compare your results with COVID-19 testing in other LMICs.

MINOR REVISIONS

9. In lines 52-53, the authors state, ‘Structural, social, epidemiological, informational, and political elements affected how publics interacted …’ Change ‘publics’ to ‘the public’

10. In line 63, the authors state, ‘….generated denial and othering narratives.’ Change ‘othering’ to ‘other’

11. In line 78, the authors state, ‘Increased availability of diagnostic interventions for COVID-19 (C-19) was….’ COVID-19 is already an acronym, so use it instead of C-19 throughout the manuscript.

12. Provide reference for lines 103-105.

13. In line 199, correct the year for Yamanis reference.

14. In line 199, you state, ‘Ha et al., 2022 and Yamanis et al., 20233 described that in areas….’ The publication years should be in brackets throughout the manuscript.

15. In line 233, provide reference for Brumwell et al. reference.

16. In line 240, the authors state, ‘C-19 was more acceptable 240 when spearheaded by people that community members…’ Add ‘testing’ after C-19.

17. Lines 254-260 is a different theme as it does not contain information on novel COVID-19 modalities.

18. Use numerical references in lines 354-360 and 379-381.

19. In line 451, the authors state, ‘The study was enhanced by the systematic searching of several databases to find all relevant studies that satisfied the predefined inclusion criteria.’ Since the authors did not search all databases, saying ‘all relevant studies’ is misleading.

Reviewer #2: This is a very relevant topic. The analysis and discussion sections were well presented. I

I suggest a review of methodology section .

• Missing, for instance, is the research question (s).

• Besides inclusion and exclusion criteria, need to explain how studies were selected, who or how many researchers conducted study selection, how conflicts were solved, if any.

• Some information in the first paragraph of results should be included in the study inclusion/exclusion, and selection.

• Lewis et al., 2021: qualitative open-ended questionnaire- is this qualitative or mixed method?

• Why is the scoping review period up to July 2023?

**********

6. PLOS authors have the option to publish the peer review history of their article (what does this mean? ). If published, this will include your full peer review and any attached files.

If you choose “no”, your identity will remain anonymous but your review may still be made public.

**Do you want your identity to be public for this peer review?** For information about this choice, including consent withdrawal, please see our Privacy Policy .

Reviewer #1: **Yes: ** Enos Moyo

Reviewer #2: **Yes: ** Emmy Kageha Igonya

**********

[NOTE: If reviewer comments were submitted as an attachment file, they will be attached to this email and accessible via the submission site. Please log into your account, locate the manuscript record, and check for the action link "View Attachments". If this link does not appear, there are no attachment files.]

While revising your submission, please upload your figure files to the Preflight Analysis and Conversion Engine (PACE) digital diagnostic tool, https://pacev2.apexcovantage.com/ . PACE helps ensure that figures meet PLOS requirements. To use PACE, you must first register as a user. Registration is free. Then, login and navigate to the UPLOAD tab, where you will find detailed instructions on how to use the tool. If you encounter any issues or have any questions when using PACE, please email PLOS at figures@plos.org . Please note that Supporting Information files do not need this step.

Attachment Submitted filename: PONE-D-24-22108 2024.docx

# Author comment: Author response to Decision Letter 0

**DOI:** 10.1371/journal.pone.0305512.r003

27 Jan 2025

Ermel Johnson, MD, MPH, PhDc

Academic Editor

PLOS ONE

22 January 2025

Subject: Rebuttal Letter for Manuscript PONE-D-24-22108

Dear Dr Ermel,

We sincerely appreciate the constructive feedback provided by the reviewers and thank you for the opportunity to revise our manuscript. Below, we provide detailed responses to each comment, addressing both major and minor revisions as requested. We believe these revisions have significantly strengthened our work.

Major Revisions

Methods

Comment 1: In the methods section, please provide information on how duplicates were removed during the search, how many researchers extracted the data independently, and how the discrepancies were handled.

Response: Thank you for raising this concern. We have revised the “Study Selection and Data Extraction” section to include these details. Specifically, duplicates were manually removed by one author, and data extraction was collaboratively conducted by three authors, enabling real-time consensus and eliminating discrepancies.

Results

Comment 2: Provide the results of the appraisal of the studies.

Response: Results of the study appraisal were included in the initial submission as Supplementary Material 2. The appraisal scores, ranging from 1 (low quality) to 4 (high quality), have been clearly outlined in the updated submission for clarity.

Comment 3: Table 3 should include a column for numerical references.

Response: Thank you for this suggestion. We have now included a column for numerical references in Table 3.

Comment 4: Key findings in Table 3 should be divided into facilitators and barriers.

Response: We appreciate this suggestion. We have now included two additional columns in Table 3: one for “Testing Facilitators” and another for “Testing Barriers.”

Comment 5: Provide a table showing themes and subthemes.

Response: A new table summarizing the themes and subthemes reported in the results section has been included as per the recommendation.

Comment 6: Authors should not rely on a single source for quotes in subthemes and should include quotes for all subthemes.

Response: Thank you for this feedback. We have now ensured that all subthemes are supported by quotes, with sources distributed across multiple studies.

Comment 7: Avoid mixing numerical referencing with APA/Harvard.

Response: The entire manuscript has been revised to use a single numerical referencing style throughout.

Discussion

Comment 8: Compare results with COVID-19 testing in other LMICs.

Response: We appreciate this suggestion and have strengthened the discussion by adding a passage (lines 513–519) that compares findings with COVID-19 testing practices in other LMICs, such as Vietnam and India. These additions highlight shared challenges, such as laboratory infrastructure deficiencies and the role of political leadership, drawing parallels to the SSA context.

Minor Revisions

Introduction and Methodology

Comment 9: Replace “publics” with “the public.”

Response: We have corrected this throughout the manuscript.

Comment 10: Replace “othering” with “other.”

Response: We appreciate the feedback but retained “othering” as it aligns with the social and psychological concept relevant to the context. The concept describes the process of perceiving or treating a person or group as different from oneself. In this context, it refers to communities or individuals perceiving other people or settings to be more vulnerable to COVID-19 and therefore requiring testing more than themselves, and refraining from testing because they perceived themselves to be at lower risk.

Comment 11: In line 78, the authors state, ‘Increased availability of diagnostic interventions for COVID-19 (C-19) was….’ COVID-19 is already an acronym, so use it instead of C-19 throughout the manuscript.

Response: The manuscript now consistently uses “COVID-19.”

Comment 12: Provide reference for lines 103-105.

Response: The requested references have been added.

Comment 13: In line 199, correct the year for Yamanis reference.

Response: Typographical errors have been addressed, and publication years are now consistently formatted in brackets throughout.

Comment 14: In line 199, you state, ‘Ha et al., 2022 and Yamanis et al., 20233 described that in areas….’ The publication years should be in brackets throughout the manuscript.

Response: Typographical errors have been addressed, and publication years are now consistently formatted in brackets throughout.

Comment 15: In line 233, provide reference for Brumwell et al. reference.

Response: The missing reference has now been added.

Comment 16: In line 240, the authors state, ‘C-19 was more acceptable 240 when spearheaded by people that community members…’ Add ‘testing’ after C-19.

Response: We have addresssed this, and ‘testing’ has now been added.

Comment 17: Lines 254-260 is a different theme as it does not contain information on novel COVID-19 modalities.

Response: A new theme, “Public Health Communication,” has been added to address this.

Comment 18: Use numerical references in lines 354-360 and 379-381.

Response: The entire manuscript has been revised to use a single numerical referencing style throughout.

Comment 19: In line 451, the authors state, ‘The study was enhanced by the systematic searching of several databases to find all relevant studies that satisfied the predefined inclusion criteria.’ Since the authors did not search all databases, saying ‘all relevant studies’ is misleading.

Response: Thank you for highlighting this important point. We agree that the original phrasing could be misleading. We have revised the statement to clarify: “The study was enhanced by the systematic searching of multiple databases to identify studies that met the predefined inclusion criteria.”

Reviewer #2

We appreciate the positive feedback regarding the relevance of the topic and the presentation of the analysis and discussion sections.

Comment 20: Missing, for instance, is the research question (s).

Response: We appreciate the feedback. Our research question, “What are the contextual factors influencing people’s decisions regarding COVID-19 testing Sub Saharan Africa?”, has been included in the final passage of the introduction section (lines ), where we have described the purpose of the study: “As part of this work, we conducted a scoping of the contextual factors influencing people’s decisions regarding COVID-19 testing in various settings throughout SSA. This information would support the implementation of the main project.”

Comment 21: Besides inclusion and exclusion criteria, need to explain how studies were selected, who or how many researchers conducted study selection, how conflicts were solved, if any.

Response: The methodology section now specifies how studies were selected, how duplicates were removed, and how consensus was reached during data extraction (lines 157-168).

Comment 22: Some information in the first paragraph of results should be included in the study inclusion/exclusion, and selection.

Response: This information has been relocated to the study inclusion/exclusion and selection subsection.

Comment 23: Lewis et al., 2021: qualitative open-ended questionnaire- is this qualitative or mixed method?

Response: We have clarified that Lewis et al. employed a qualitative approach using an asynchronous opened-ended online questionnaire, where respondents recorded their answers to pre-arranged questions, and responses from purposively sampled diagnostic radiographers were thematically analysed.

Comment 24: Why is the scoping review period up to July 2023?

Response: The scoping review period was strategically set to follow the global peak of the pandemic when a substantial body of research on COVID-19 testing was anticipated.

We have carefully addressed all reviewer comments to enhance the manuscript's clarity, rigor, and relevance. We trust that these revisions meet the expectations of the reviewers and the journal.

Thank you once again for the opportunity to revise and resubmit our manuscript. We look forward to your feedback.

Yours sincerely,

Mackwellings Phiri

(On behalf of authors)

Attachment Submitted filename: Response to Reviewers.docx

# Editor report: Decision Letter 1

**DOI:** 10.1371/journal.pone.0305512.r004

**Authors:** Ermel Johnson

7 Feb 2025

Factors influencing uptake of COVID-19 diagnostics in Sub-Saharan Africa: a rapid scoping review

PONE-D-24-22108R1

Dear Dr. Phiri,

We’re pleased to inform you that your manuscript has been judged scientifically suitable for publication and will be formally accepted for publication once it meets all outstanding technical requirements.

Within one week, you’ll receive an e-mail detailing the required amendments. When these have been addressed, you’ll receive a formal acceptance letter and your manuscript will be scheduled for publication.

An invoice will be generated when your article is formally accepted. Please note, if your institution has a publishing partnership with PLOS and your article meets the relevant criteria, all or part of your publication costs will be covered. Please make sure your user information is up-to-date by logging into Editorial Manager at Editorial Manager®  and clicking the ‘Update My Information' link at the top of the page. If you have any questions relating to publication charges, please contact our Author Billing department directly at authorbilling@plos.org.

If your institution or institutions have a press office, please notify them about your upcoming paper to help maximize its impact. If they’ll be preparing press materials, please inform our press team as soon as possible -- no later than 48 hours after receiving the formal acceptance. Your manuscript will remain under strict press embargo until 2 pm Eastern Time on the date of publication. For more information, please contact onepress@plos.org.

Kind regards,

Ermel Johnson, MD, MPH, PhDc

Academic Editor

PLOS ONE

Additional Editor Comments:

We congratulate and appreciate the efforts made by authors to resolve the revision suggested by the reviewers. the manuscript is in good shape. however, minors adjustments need to be made:

- remove empty lines like 93, 109, 115, 191, .... although the manuscript

- specify in the table 1 (line 159) if you consider the preprints articles as inclusion or exclusion criteria or not. It will help readers to know if they were used or not.

- line 183, change "employed" by "used" or "adopted"

- in response to comment 5, you said "A new table summarizing the themes and subthemes reported in the results section has been included as per the recommendation". However, the table is the methods section. I agree that the table should be in the data analysis sub-section

- the response to the comment 24, is it correct that the scoping review was conducting from July to August 2023, not the date of publication of papers include? I was to be sure.

# Editor report: Acceptance letter

**DOI:** 10.1371/journal.pone.0305512.r005

**Authors:** Ermel Johnson

PONE-D-24-22108R1

PLOS ONE

Dear Dr. Phiri,

I'm pleased to inform you that your manuscript has been deemed suitable for publication in PLOS ONE. Congratulations! Your manuscript is now being handed over to our production team.

At this stage, our production department will prepare your paper for publication. This includes ensuring the following:

* All references, tables, and figures are properly cited

* All relevant supporting information is included in the manuscript submission,

* There are no issues that prevent the paper from being properly typeset

If revisions are needed, the production department will contact you directly to resolve them. If no revisions are needed, you will receive an email when the publication date has been set. At this time, we do not offer pre-publication proofs to authors during production of the accepted work. Please keep in mind that we are working through a large volume of accepted articles, so please give us a few weeks to review your paper and let you know the next and final steps.

Lastly, if your institution or institutions have a press office, please let them know about your upcoming paper now to help maximize its impact. If they'll be preparing press materials, please inform our press team within the next 48 hours. Your manuscript will remain under strict press embargo until 2 pm Eastern Time on the date of publication. For more information, please contact onepress@plos.org.

If we can help with anything else, please email us at customercare@plos.org.

Thank you for submitting your work to PLOS ONE and supporting open access.

Kind regards,

PLOS ONE Editorial Office Staff

on behalf of

Dr. Ermel Johnson

Academic Editor

PLOS ONE
